# Circulating inflammatory cytokines and the risk of myasthenia gravis: a bidirectional Mendelian randomization study

**DOI:** 10.1186/s12883-025-04271-9

**Published:** 2025-07-01

**Authors:** Boyang Su, Zhengqing He, Luyao Shi, Mao Li, Xusheng Huang

**Affiliations:** 1 Medical School of Chinese PLA, Beijing, China; 2 Neurological Department of the First Medical Center, Chinese PLA General Hospital, No. 28 Fuxing Road, Haidian District 100853, Beijing, China; 3 Department of Neurology, Beijing Friendship Hospital, Capital Medical University, Beijing, China; 4 Department of Gastrointestinal Oncology, Harbin Medical University Cancer Hospital, Harbin, China

**Keywords:** Circulating inflammatory cytokines, Myasthenia gravis, Bidirectional, Mendelian randomization, Instrumental variable (IV)

## Abstract

**Background:**

Myasthenia gravis (MG) is an autoimmune disorder of the neuromuscular junction. Increasing evidence has suggested inflammation is involved in the pathogenesis of MG, but whether it is the cause or a downstream effect remains unclear. In this study, a two-sample Mendelian randomization (TSMR) analysis was performed to explore the causal relationship between 91 circulating inflammatory cytokines and MG.

**Method:**

In this study, the data of 91 circulating inflammatory cytokines from 4824 Europeans and the largest GWAS database of MG (1873 patients and 36370 controls) were used to screen instrumental variables (IVs). Inverse variance weighting (IVW), Bayesian weighted MR (BWMR), MR-Egger regression, weighted median (WM), simple mode and weighted mode were used to evaluate the association between MG and inflammatory cytokines. The MR-Egger intercept test and Cochran’s Q test were used to test the pleiotropy and heterogeneity of IVs.

**Result:**

Our results showed that adenosine deaminase (ADA) and CD40 Ligand‌ (CD40L) are positively associated with the risk of MG (OR = 1.16, 95%CI: 1.00-1.33, *P* = 0.041; OR = 1.20, 95%CI: 1.02–1.40, *P* = 0.025), while interleukin-1-alpha (IL-1α), glial-cell-line-derived neurotrophic factor (GDNF), Osteoprotegerin (OPG) and tumor necrosis factor-beta (TNF-β) are negatively associated with the risk of MG (OR = 0.80, 95% CI: 0.64 ~ 0.99, *P* = 0.042; OR = 0.74, 95%CI:0.58 ~ 0.0.96, *P* = 0.022; OR = 0.76, 95% CI: 0.61 ~ 0.94, *P* = 0.013; OR = 0.76, 95% CI: 0.61 ~ 0.94, *P* = 0.012; OR = 0.80, 95% CI: 0.68 ~ 0.93, *P* = 0.006). In addition, genetically predicted MG affected the expression of seven cytokines. Sensitivity analysis showed no horizontal pleiotropy and significant heterogeneity of all results.

**Conclusions:**

Our results provided promising clues for the treatment of MG. We evaluated the association between inflammatory cytokines and the disease by genetic informatics approach, which may help to better understand the underlying mechanisms of MG.

**Supplementary Information:**

The online version contains supplementary material available at 10.1186/s12883-025-04271-9.

## Introduction

Myasthenia gravis (MG) is an autoimmune disease characterized by fluctuating muscle weakness and abnormal fatigue [1]. The lesion is located on the postsynaptic membrane of the neuromuscular junction. After being attacked by autoantibodies, the number of acetylcholine receptors on the postsynaptic membrane decreases [1]. The global prevalence of MG is estimated to be between 150 and 250 per million, with a higher incidence in females than in males [2]. Although current treatment methods are well-established, there are still some patients with poor efficacy. Inflammatory targets is an important adjuvant therapy to MG [3].

Inflammation is mainly mediated by cytokines which are released into the peripheral circulation mainly by monocytes and macrophages [4]. MG is an acquired autoimmune disease, and the progression of disease is closely related to inflammatory cytokines [5]. Previous studies indicated that infiltrated macrophages and monocytes at the neuromuscular junctions of MG patients release various inflammatory cytokines, recruiting immune cells that penetrate specific sites and initiate inflammatory responses [6]. The thymus plays a major role in anti-acetylcholine receptor (AChR) antibody-mediated MG, and up-regulation of interleukin-6 (IL-6) and transforming growth factor-β (TGF-β) is found in thymic epithelial cells of MG patients [7]. Moreover, interferon-β (IFN-β) induces the expression of α-AChR in thymic epithelial cells and increases the expression of chemokines, leading to the formation of thymic germinal centers and abnormal aggregation of peripheral immune cells [8]. Most studies have focused on the effects of specific inflammatory cytokines released by immune organs in patients with MG, while changes in systemic circulating inflammatory cytokines are equally important [9–11]. Finally, the results of observational studies are prone to be affected by acquired confounding factors, it is difficult to establish a clear relationship.

Mendelian randomization (MR) is a causal inference method grounded in genetic variations, employing genetic variants as instrumental variables (IVs) to elucidate causal associations between exposures and outcomes within non-experimental datasets [12]. MR leverages the stability of genes and Mendelian laws of inheritance; whereby parental alleles are randomly distributed to offspring. The relationship between genes and outcomes remains impervious to confounding factors, ensuring the integrity of causal inference [13]. In this study, two-sample MR analysis was used to evaluate the association between circulating inflammatory cytokines and MG.

## Method

### MR and assumptions

**Fig. 1:**
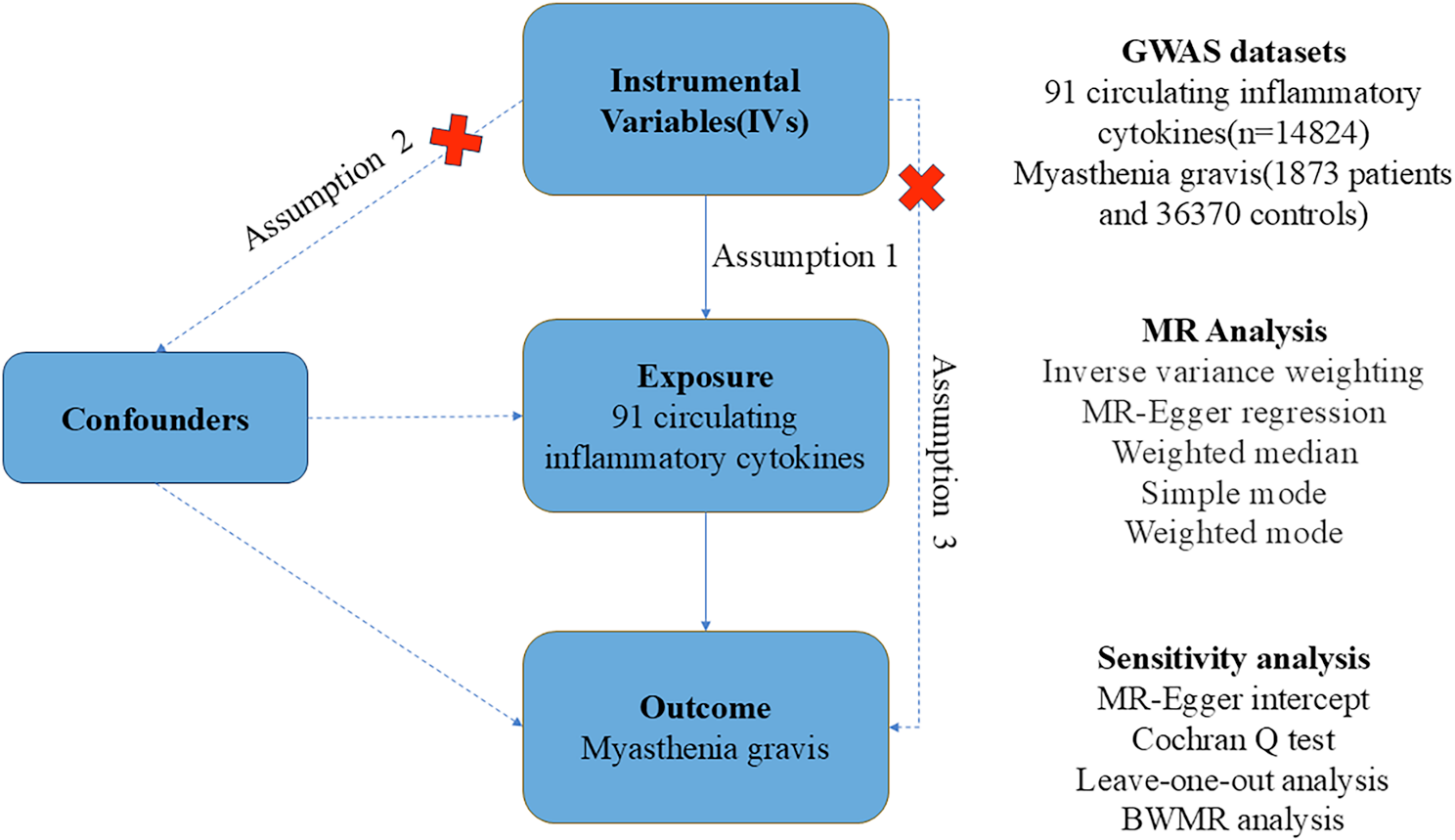
An overall design of the present study. Abbreviations: GWAS, genome-wide association study; MR, Mendelian randomization; BWMR, Bayesian Weighted Mendelian Randomization Analysis

Figure 1 shows the design of our TSMR study (Fig. 1). In this study, genetic variants of 91 circulating inflammatory cytokines were used as IVs and bidirectional MR was used to analyze the causal relationship between these cytokines and MG. MR was based on the following three assumptions [14]: [1] Relevance Assumption: Instrumental variables were strongly correlated with exposure factors [2]. Independence Assumption: Instrumental variable was not associated with any possible confounding factors [3]. Exclusion Restriction Assumption: Instrumental variables only have an effect on disease outcomes through the exposure factors studied, and do not directly affect disease outcomes through other pathways or indirectly affect disease outcomes through other pathways.

### Circulating inflammatory cytokines

The summary data for 91 circulating inflammatory cytokines were derived from a GWAS database, the study conducted a genome-wide protein quantitative trait locus (pQTL) study of 14,824 European-ancestry participants and measured 91 plasma proteins using the Olink Target platform [15].

### Myasthenia gravis

The summary data for MG were obtained from GWAS database in the United States and Italy, involving blood samples from 1,873 patients diagnosed with AChR antibody-positive myasthenia gravis and 36,370 healthy individuals [16]. The study included only patients with AChR + MG and excluded those with muscle-specific kinase antibody positive (MuSK+). MG diagnosis was based on the standard clinical criteria of fatigable weakness and electrophysiological and/or pharmacological anomalies [16]. Institutional review board endorsements were secured from all institutions involved in the study.

### Instrumental variable selection

We set *P* < 5 × 10^− 8^ as the genome-wide significant threshold to select strongly associated SNPs with MG and inflammatory cytokines. We found only 4 SNPs for cytokines at *P* < 5 × 10^− 8^. Therefore, a significance threshold (*P* < 5 × 10^− 6^) was used to select instrumental variables, which could better represent the genetic variation of exposure factors and effectively evaluate the association between exposure and outcome in the subsequent inference process. (Supplementary Table 1, 2). To remove linkage disequilibrium, we set the screening condition (r^2^ = 0.001, kb = 10000) to ensure that the choice of IVs was independent of each other. Finally, the weak instrumental variables were removed using F-statistic>10 as the criterion (Supplementary Table 1, 2).

## Statistical analyses

### Two-sample MR analysis

TSMR Analysis was performed in this experiment using five main methods: inverse variance weighting (IVW), MR-Egger regression, weighted median (WM), simple mode, and weighted mode. The primary analysis method was IVW to evaluate the presupposed SNPs as valid IVs and the ratio method was used to calculate the single SNP to obtain the estimate under the premise of no horizontal pleiotropy, thereby precisely estimating the aggregate causal effect between exposure and outcome [17]. MR-Egger regression and the WM were complementary to the IVW method. The MR-Egger regression method needed to satisfy the InSIDE assumption (instrument strength independent of direct effect assumption), which weakens the exclusivity assumption in the IVW method. The WM assumed that there is pleiotropy in less than 50% of SNPs and the median of the distribution function obtained by ordering the effect sizes of all individual SNPs by weight [18]. This study protocol and details are not pre-registered.

### Bayesian weighted Mendelian randomization analysis

Bayesian Weighted Mendelian Randomization Analysis (BWMR) was performed to further verify the results. BWMR effectively use the information of instrumental variables to balance the differences between the instrumental variables, reduce the influence caused by the instability of individual instrumental variables, and improve the accuracy and stability of causal effect estimation [19]. BWMR was performed using ‘BWMR’ package in R software (Version 4.3.0).

### Sensitivity analysis

Statistical heterogeneity among SNPs was assessed by Cochran Q test, and *P* < 0.05 was considered statistically significant. MR-Egger intercept term was used to test whether there was gene pleiotropy between circulating inflammatory cytokines and MG. If the intercept was different from zero, it indicated that there was horizontal pleiotropy in the study, otherwise there was not exist. Leave-one-out analysis was used to gradually remove SNPs, calculate the combined effect of the remaining SNPs and observe the influence of each SNP on the results to determine the degree of SNP influence on the results. All methods used in this study were analyzed using the TwoSampleMR package in R software (Version 4.3.0) [20].

## Results

### MR analysis for effect of 91 Circulating inflammatory cytokines on MG

**Fig. 2:**
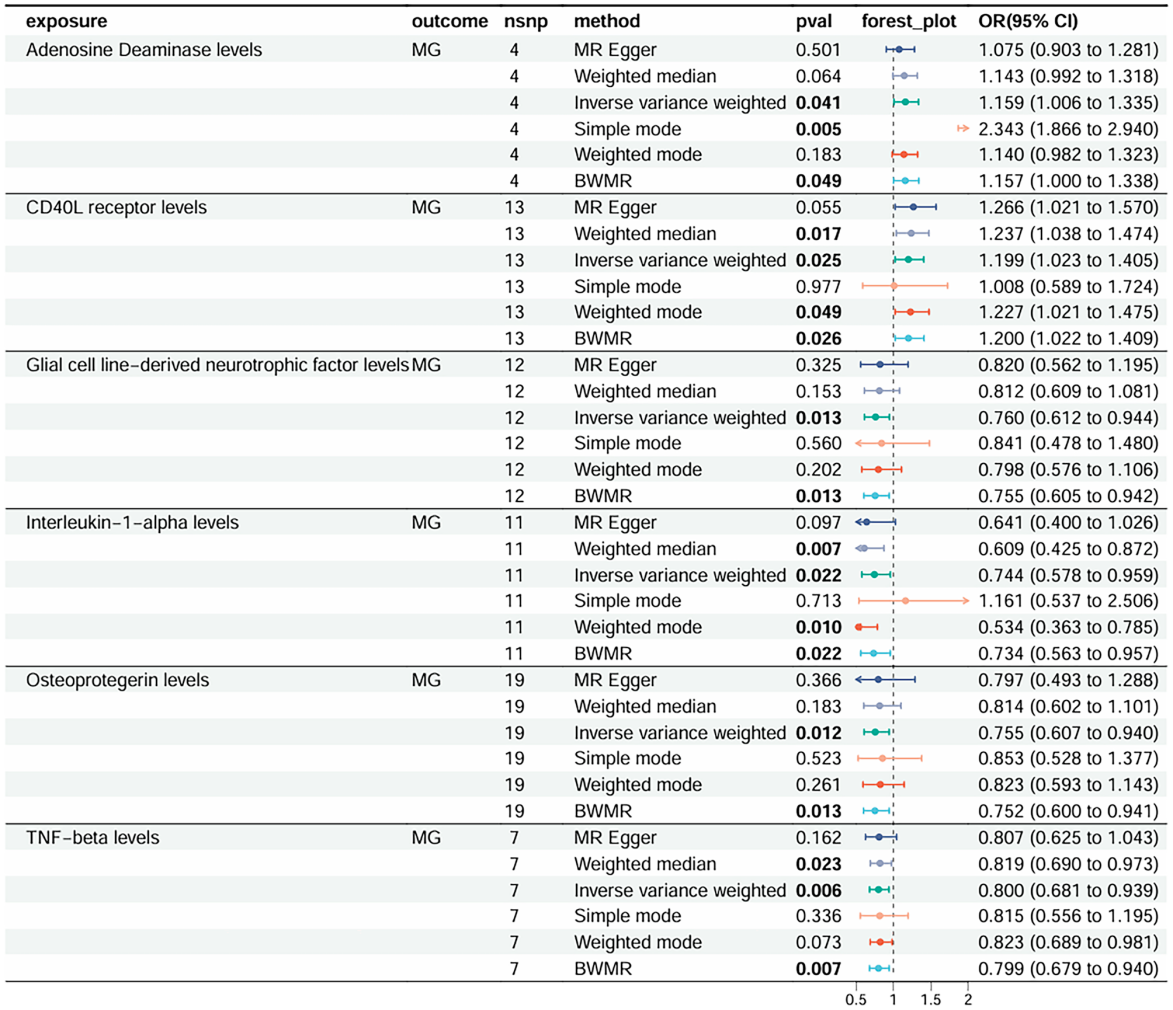
Forest plot of the MR analyses for the associations between 91 circulating inflammatory cytokines and risk of MG. Odds ratios (OR) and 95% confidence intervals (CI) represent the change in odds ratios of myasthenia gravis with each 1-SD increase in inflammatory cytokine levels

The effect of 91 circulating inflammatory cytokines on the risk of MG is shown in the forest plot (Fig. 2). The results of IVW showed that six inflammatory cytokines were associated with the risk of MG, including adenosine deaminase (ADA), CD40 Ligand‌ (CD40L), interleukin-1-alpha (IL-1α), glial-cell-line-derived neurotrophic factor (GDNF), Osteoprotegerin (OPG) and tumor necrosis factor-beta (TNF-β) (Supplementary Table 3, Fig. 2). Genetically predicted ADA and CD40L were positively correlated with the risk of MG, indicating that the levels of these two inflammatory cytokines have adverse effects on MG. For each 1-standard deviation (SD) increase in ADA (OR = 1.16, 95%CI: 1.00-1.33; *P* = 0.041) and CD40L receptor (OR = 1.20, 95%CI: 1.02–1.40; *P* = 0.025) levels, the risk of MG increased by 16% and 20%, respectively. In addition, IL-1α (OR = 0.74, 95%CI:0.58 ~ 0.0.96;*P* = 0.022), GDNF (OR = 0.76, 95% CI: 0.61 ~ 0.94; *P* = 0.013), OPG (OR = 0.76, 95% CI: 0.61 ~ 0.94; *P* = 0.012) and TNF-β (OR = 0.80, 95% CI: 0.68 ~ 0.93; *P* = 0.006) were negatively associated with the risk of MG. MR-Egger analysis showed that higher IL-1α was associated with the lower risk of MG, and for each 1-SD increase, the risk of MG as reduced by 31% (OR = 0.61; 95%CI: 0.39–0.96; *P* = 0.045). The funnel plots and scatter plots are shown in Supplementary Fig. 1–2. We used the BWMR method to validate our findings, which demonstrated that the above six inflammatory cytokines were associated with a high risk of MG (Supplementary Table 9).

### Reverse MR analysis

**Fig. 3:**
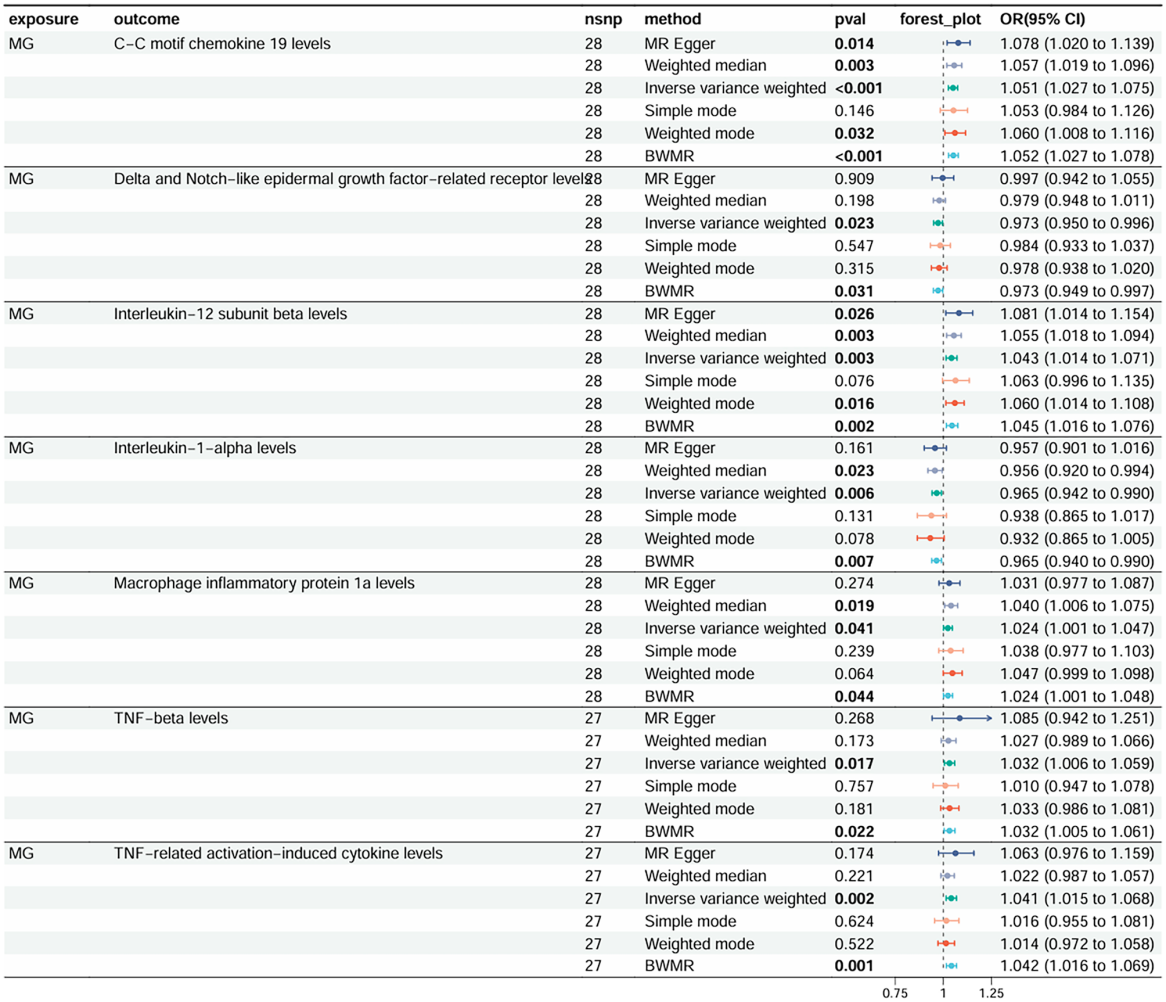
Forest plot of reverse MR analysis of the relationship between 91 circulating inflammatory cytokines and the risk of MG

The results of reverse MR analysis of MG and 91 circulating inflammatory cytokines are shown in the forest plot (Fig. 3, Supplementary Table 4). The risk of MG was found to be positively associated with C-C motif chemokine 19 (CCL19) (OR = 1.05, 95%CI:1.02 ~ 1.07; *P*<0.001), TNF-related activation-induced cytokine (TRANCE) (OR = 1.04, 95%CI:1.02 ~ 1.07;*P* = 0.006), TNF-β (OR = 1.03, 95%CI:1.00 ~ 1.06;*P* = 0.017), macrophage inflammatory protein 1a (MIP-1α) (OR = 1.02, 95%CI:1.00 ~ 1.05;*P* = 0.04), interleukin-12 subunit beta (IL-12β) (OR = 1.04, 95%CI:1.01 ~ 1.07;*P* = 0.002), and negatively associated with Delta and Notch-like epidermal growth factor-related receptor (DNER)(OR = 0.97, 95%CI:0.95 ~ 1.00; *P* = 0.02) and IL-1α (OR = 0.96, 95%CI:0.94 ~ 0.99;*P* = 0.006) (Supplementary Fig. 5). The funnel plots and scatter plots are shown in Supplementary Fig. 4–5. We used the BWMR method to validate our findings, which demonstrated that the above seven inflammatory cytokines were associated with a high risk of MG (Supplementary Table 10).

### Sensitivity analyses

The MR-Egger intercept test and the Cochran Q test found no significant horizontal pleiotropy or heterogeneity among these inflammatory cytokines (Supplementary Tables 5–8). Leave-one-out analysis indicated that no single SNP significantly affected the results of MR (Supplementary Figs. 3 and 6).

## Discussion

In this study, the genetic data of 91 circulating inflammatory cytokines were used as exposure cytokines to explore the relationship with the risk of MG. The results showed that ADA and CD40L were positively associated with the risk of MG and IL-1α, GDNF, OPG and TNF-β were negatively associated with the risk of MG. Reverse MR analysis showed that the risk of MG was associated with CCL19, TRANCE, IL-12β, TNF-β, MIP-1α, IL-1β and DNER. There is a bidirectional causal relationship between IL-1α and TNF-β and the risk of MG, which may be located in the upstream and downstream of MG progression.

Inflammation plays an important role in the pathogenesis of MG. Previous studies have reported that the level of interleukin-36γ in MG patients is positively associated with the severity of the disease and may be a serological marker of MG [21]. Moreover, with the increase of interleukin-33 and interleukin-17α levels, the quantitative MG score tended to increase [22]. The increased expression of C-X-C motif chemokine 13 in thymic epithelial cells caused the recruitment of B lymphocytes in MG model mice, which up-regulated the level of anti-AChR [23]. These studies have shown that circulating inflammatory cytokines are closely related to the pathogenesis of MG. However, observational studies are subject to confounding bias. In this study, two-way MR analysis was performed to determine the upstream and downstream circulating inflammatory cytokines of MR.

Adenosine acts on receptors on the surface of immune cells in the immune microenvironment to regulate the activity, proliferation and differentiation of immune cells [24]. Previous studies have shown that administration of ADA at the early stage of MG reduces clinical symptom scores and the number of inflammatory cell infiltration at the neuromuscular junction in MG model mice [25]. In addition, the activity of ADA in serum of MG patients was positively correlated with the aggravation of MG, and ADA was involved in the pathogenesis of MG by changing the function of peripheral blood lymphocytes [26]. ADA converted adenosine to inosine, which relieved the inhibitory effect of adenosine on the release of acetylcholinesterase (AChE) and increased the release of AChE. Acetylcholine was rapidly broken down by AChE at the neuromuscular junction, resulting in a decrease in acetylcholine, which affects the transmission of nerve impulses to the muscle, thereby aggravating the symptoms of muscle weakness [27]. Our study showed that ADA increased the risk of MG. It is speculated that ADA can be used as a biomarker of MG and can be used to evaluate the severity of MG.

CD40L is expressed on the surface of activated T cells and can bind to CD40 on B cells to affect the proliferation and differentiation of B cells. In MG patients, the activation of CD40L signaling pathway promotes the maturation of autoreactive B cells and the production of antibodies, especially the production of autoantibodies against AChR and MuSK antigens. These autoantibodies are one of the main factors leading to the pathological changes of MG [28–30]. The results showed that the mRNA expression level of CD40L in peripheral blood CD4 + T cells of AChR-MG and MuSK-MG patients was decreased compared with that of healthy controls [31]. In addition to T cells, other immune cells in peripheral blood, such as monocytes, can also express CD40L. However, there are few studies on the expression level of non-T cell-derived CD40L in peripheral blood of MG patients. Some studies have found that the expression level of CD40L in peripheral blood monocytes of patients with systemic lupus erythematosus is related to disease activity [32]. Our results showed that increased CD40L could promote the risk of MG, suggesting that peripheral blood CD40L may be involved in the disease process in autoimmune diseases, but its role in MG patients needs further study. Furthermore, Our study showed that the increased levels of GNDF, and OPG were cytokines that reduce the risk of MG. At present, there are few studies on these inflammatory cytokines and MG, and the mechanism of action on MG needs to be further elucidated. In 1994, the thymus of MG patients were found to secrete large amounts of interleukin, which activate a series of inflammatory responses by stimulating T cells [33]. In addition, IL-1α was a disease modifier of MG and was associated with early onset MG [34]. Liu et al. found that IL-1 levels were significantly increased in the brain, thymus and blood of MG rats [35]. In addition, it has been shown that IL-1α binds to receptors on the surface of immune cells and promotes the proliferation and differentiation of T and B cells. In autoimmune diseases, IL-1 enhanced the activity of autoreactive T cells and B cells, assisting B cells to produce antibodies against autoantigens [36]. Furthermore, IL-1α promoted the activation and function of Treg cells, thereby inhibiting the excessive activation of autoimmune responses [36]. Our study found that IL-1α was negatively associated with the risk of MG. We speculate that this is related to the bidirectional immunomodulation of IL-1α. There are few studies on TNF-β and MG. The cytokine TNF-β, secreted by T lymphocytes, elicits inflammation and induces cell death upon binding to tumor necrosis factor receptor (TNFR1) [37]. TNF-β and TNFRp55 knockout mice exhibited resistance to EAMG, suggesting that TNF may exacerbate EAMG symptoms [38]. In contrast, Manz et al. found no difference in TNF-β allele frequency in MG patients compared with healthy controls [39]. The reverse association of TNF-β with the risk of MG in our study contradicts previous findings, and we suggested that it may be due to confounding bias in observational experiments or different genetic associations among different populations. There is no research on the relationship between DNFR and OPG and MG. In our study, these two inflammatory cytokines were negatively associated with the risk of MG, and the mechanism of action needs to be further explored.

Many studies have indicated that the levels of inflammatory cytokines in MG patients are elevated [40–42]. In our study, the reverse MR analysis revealed a group of circulating inflammatory cytokines levels increased by MG, including CCL19, TRANCE, IL-12β, TNF-β, MIP-1α, IL-1α and DNER. This suggests that further exploration of the connection between MG and circulating inflammatory cytokines is valuable. Further large-scale clinical or animal experiments are required for verification.

The targeted therapy of inflammatory cytokines for MG has been studied previously. The treatment of EAMG mice with recombinant human tumor necrosis factor receptor soluble protein (TNFR) Fc blocking endogenous TNF can relieve the symptoms of asthenia, but cannot inhibit the serum anti-AChR antibodies [38]. In addition, tocilizumab is a recombinant humanized anti-human interleukin-6 receptor monoclonal antibody, which showed significant efficacy in refractory MG patients [43]. complement C3 activation has been proven to form membrane attack complex (MAC) on the cell membrane and participate in the destruction of neuromuscular junction [44]. Treatment with IL-1 receptor antagonist (IL-1ra) reduced C3 levels in MG model mice, reduced muscle AChR loss, and improved the symptoms of myasthenia mice. Therefore, some researchers speculated that the combination of IL-1 receptor antagonist and other anti-inflammatory factor targeted agents may be more beneficial to the relief of clinical symptoms of MG [45]. However, some exploratory targeted antagonists targeting inflammatory cytokines have not shown good effects. For example, etanercept (an antagonist of TNF-α) was only effective in a subset of MG patients [43] and aggravates MG symptoms in rheumatoid arthritis patients [46]. In addition, humanized monoclonal antibodies against interferon-α, such as ronalizumab and anifumab, have had conflicting results in mouse models of MG [3]. In our study, these two inflammatory cytokines were not related to the risk of MG, so we speculate that this may be the reason for the poor effect of treatment and the inability to effectively prevent the progression of the disease. At present, the research on targeted therapy for inflammation is not in-depth, and some drugs have poor efficacy, short duration of efficacy, and easy to cause side effects. More high-quality clinical studies are needed to confirm the efficacy and safety of these drugs in the future.

This is the first Mendelian randomized study to evaluate the causal relationship between MG and 91 inflammatory cytokines. Our study has some limitations. Firstly, since all MG patients were from Italy and the United States, this study may not be available in other ethnic groups, so further exploration of applicability to other ethnic groups is required. Secondly, we have lowered the threshold standard for screening IVs, but we still could not be completely excluded the influence of weak instrument effect on the outcome. In the future, researchers need to expand the sample size of MG and include different ethnic groups for research. Thirdly, we considered that AchR MG is a heterogeneous population and that the severity and course of the disease as well as the use of immunosuppressive agents may alter the levels of inflammatory cytokines. A large number of observational studies are needed to confirm our results. Overall, the results of our MR analysis were not similar to those of some previous clinical trials, which we analyzed may be related to the different stages of the disease or various different cytokines in life activities.

## Conclusion

In this study, we have demonstrated a bidirectional causal relationship between MG and 91 inflammatory cytokines using MR Methods. We found that six inflammatory cytokines significantly associated with MG were positively verified, and the reverse MR analysis revealed seven inflammatory cytokines that were associated. These correlation results need to be interpreted with caution, which provides a valuable direction for further research on the potential etiology and inflammatory pathways of MG.

## Electronic supplementary material

Below is the link to the electronic supplementary material.

Supplementary Material 1

Supplementary Material 2

Supplementary Material 3

Supplementary Material 4

Supplementary Material 5

Supplementary Material 6

Supplementary Material 7

Supplementary Material 8

## Data Availability

No datasets were generated or analysed during the current study.
