# Supplementary material for: Circulating inflammatory cytokines and the risk of myasthenia gravis: a bidirectional Mendelian randomization study
Source: BMC Neurol. 2025 Jul 1;25:271. doi: 10.1186/s12883-025-04271-9 (PMC12211973; doi:10.1186/s12883-025-04271-9)
Supplement: Supplementary file 1 — Supplementary Material 1 [file 12883_2025_4271_MOESM1_ESM.pdf]

STROBE-MR checklist of recommended items to address in reports of Mendelian randomization studies

Item No.	Section	Checklist item	Page No.	Relevant text from manuscript
1	**TITLE and ABSTRACT**	Indicate Mendelian randomization (MR) as the study's design in the title and/or the abstract if that is a main purpose of the study	1	Circulating inflammatory cytokines and the risk of Myasthenia gravis: a bidirectional Mendelian randomization study / In this study, a two-sample Mendelian randomization (TSMR) analysis was performed to explore the causal relationship between 91 circulating inflammatory cytokines and MG.
	**INTRODUCTION**			
2	**Background**	Explain the scientific background and rationale for the reported study. What is the exposure? Is a potential causal relationship between exposure and outcome plausible? Justify why MR is a helpful method to address the study question	4	MR leverages the stability of genes and Mendelian laws of inheritance, whereby parental alleles are randomly distributed to offspring. The relationship between genes and outcomes remains impervious to confounding factors, ensuring the integrity of causal inference. In this study, two-sample MR analysis was used to evaluate the causal relationship between circulating inflammatory factors and MG./ In this study, two-sample MR analysis was used to evaluate the association between circulating inflammatory cytokines and MG.
3	**Objectives**	State specific objectives clearly, including pre-specified causal hypotheses (if any). State that MR is a method that, under specific assumptions, intends to estimate causal effects	4	MR leverages the stability of genes and Mendelian laws of inheritance, whereby parental alleles are randomly distributed to offspring. The relationship between genes and outcomes remains impervious to confounding factors, ensuring the integrity of causal inference. In this study, two-sample MR analysis was used to evaluate the causal relationship between circulating inflammatory factors and MG./ In this study, two-sample MR analysis was used to evaluate the association between circulating inflammatory cytokines and MG.
	**METHODS**			
4	**Study design and data sources**	Present key elements of the study design early in the article. Consider including a table listing sources of data for all phases of the study. For each data source contributing to the analysis, describe the following:	4	Figure 1 shows the design of our TSMR study (Figure 1).

	a)	Setting: Describe the study design and the underlying population, if possible. Describe the setting, locations, and relevant dates, including periods of recruitment, exposure, follow-up, and data collection, when available.	5	The summary data for 91 circulating inflammatory cytokines were derived from a GWAS database, the study conducted a genome-wide protein quantitative trait locus (pQTL) study of 14824 participants and measured 91 plasma proteins using the Olink Target platform. / The summary data for MG were obtained from GWAS database in the United States and Italy, involving blood samples from 1,873 patients diagnosed with acetylcholine receptor (AChR) antibody-positive myasthenia gravis and 36,370 healthy individuals (16). The study included only patients with AChR+ MG and excluded those with muscle-specific kinase antibody positive (MuSK+). MG diagnosis was based on the standard clinical criteria of fatigable weakness and electrophysiological and/or pharmacological anomalies(16).
	b)	Participants: Give the eligibility criteria, and the sources and methods of selection of participants. Report the sample size, and whether any power or sample size calculations were carried out prior to the main analysis	5	The summary data for 91 circulating inflammatory cytokines were derived from a GWAS database, the study conducted a genome-wide protein quantitative trait locus (pQTL) study of 14824 participants and measured 91 plasma proteins using the Olink Target platform. / The summary data for MG were obtained from GWAS database in the United States and Italy, involving blood samples from 1,873 patients diagnosed with acetylcholine receptor (AChR) antibody-positive myasthenia gravis and 36,370 healthy individuals (16). The study included only patients with AChR+ MG and excluded those with muscle-specific kinase antibody positive (MuSK+). MG diagnosis was based on the standard clinical criteria of fatigable weakness and electrophysiological and/or pharmacological anomalies(16).
	c)	Describe measurement, quality control and selection of genetic variants	6	We set $P<5\times10-8$ as the genome-wide significant threshold to select strongly associated SNPs with MG and inflammatory cytokines. We found only 4 SNPs for cytokines at $P<5\times10-8$. Therefore, a significance threshold ($P<5\times10-6$) was used to select instrumental variables, which could better represent the genetic variation of exposure factors and effectively evaluate the association between exposure and outcome in the subsequent inference process. (Supplementary Table 1、2). To remove linkage disequilibrium, we set the screening

					condition (r2=0.001, kb=10000) to ensure that the choice of IVs was independent of each other. Finally, the weak instrumental variables were removed using F-statistic＞10 as the criterion (Supplementary Table 1、2).
		d)	For each exposure, outcome, and other relevant variables, describe methods of assessment and diagnostic criteria for diseases	5	The summary data for MG were obtained from GWAS database in the United States and Italy, involving blood samples from 1,873 patients diagnosed with acetylcholine receptor (AChR) antibody-positive myasthenia gravis and 36,370 healthy individuals (16). The study included only patients with AChR+ MG and excluded those with muscle-specific kinase antibody positive (MuSK+). MG diagnosis was based on the standard clinical criteria of fatigable weakness and electrophysiological and/or pharmacological anomalies(16). Institutional review board endorsements were secured from all institutions involved in the study.
		e)	Provide details of ethics committee approval and participant informed consent, if relevant	5	Institutional review board endorsements were secured from all institutions involved in the study.
5	**Assumptions**		Explicitly state the three core IV assumptions for the main analysis (relevance, independence and exclusion restriction) as well assumptions for any additional or sensitivity analysis	4	MR was based on the following three assumptions(14): (1) Relevance Assumption: Instrumental variables were strongly correlated with exposure factors. (2) Independence Assumption: Instrumental variable was not associated with any possible confounding factors. (3) Exclusion Restriction Assumption: Instrumental variables only have an effect on disease outcomes through the exposure factors studied, and do not directly affect disease outcomes through other pathways or indirectly affect disease outcomes through other pathways.
6	**Statistical methods: main analysis**		Describe statistical methods and statistics used		
		a)	Describe how quantitative variables were handled in the analyses (i.e., scale, units, model)	6	We set P<5×10-8 as the genome-wide significant threshold to select strongly associated SNPs with MG and inflammatory cytokines. We found only 4 SNPs for cytokines at P<5×10−8. Therefore, a significance threshold (P<5×10−6) was used to select instrumental variables, which could better represent the genetic variation of exposure factors

				and effectively evaluate the association between exposure and outcome in the subsequent inference process. (Supplementary Table 1、2). To remove linkage disequilibrium, we set the screening condition (r2=0.001, kb=10000) to ensure that the choice of IVs was independent of each other. Finally, the weak instrumental variables were removed using F-statistic＞10 as the criterion (Supplementary Table 1、2).
	b)	Describe how genetic variants were handled in the analyses and, if applicable, how their weights were selected	6	We set P<5×10-8 as the genome-wide significant threshold to select strongly associated SNPs with MG and inflammatory cytokines. We found only 4 SNPs for cytokines at P<5×10−8. Therefore, a significance threshold (P<5×10−6) was used to select instrumental variables, which could better represent the genetic variation of exposure factors and effectively evaluate the association between exposure and outcome in the subsequent inference process. (Supplementary Table 1、2). To remove linkage disequilibrium, we set the screening condition (r2=0.001, kb=10000) to ensure that the choice of IVs was independent of each other. Finally, the weak instrumental variables were removed using F-statistic＞10 as the criterion (Supplementary Table 1、2). / TSMR Analysis was performed in this experiment using five main methods: inverse variance weighting (IVW), MR-Egger regression, weighted median (WM), simple mode, and weighted mode. Initially, the IVW method was used to evaluate the presupposed SNPs as valid IVs and the ratio method was used to calculate the single SNP to obtain the estimate under the premise of no horizontal pleiotropy, thereby precisely estimating the aggregate causal effect between exposure and outcome. MR-Egger regression and the WM were complementary to the IVW method. The MR-Egger regression method needed to satisfy the InSIDE (instrument strength independent of direct effect assumption, which weakens the exclusivity assumption in the IVW method. The WM assumed that there is pleiotropy in less than 50% of SNPs and the median of the distribution function obtained by ordering the effect sizes of all individual SNPs by weight.

		c)	Describe the MR estimator (e.g. two-stage least squares, Wald ratio) and related statistics. Detail the included covariates and, in case of two-sample MR, whether the same covariate set was used for adjustment in the two samples	6	TSMR Analysis was performed in this experiment using five main methods: inverse variance weighting (IVW), MR-Egger regression, weighted median (WM), simple mode, and weighted mode. The primary analysis method was IVW to evaluate the presupposed SNPs as valid IVs and the ratio method was used to calculate the single SNP to obtain the estimate under the premise of no horizontal pleiotropy, thereby precisely estimating the aggregate causal effect between exposure and outcome. MR-Egger regression and the WM were complementary to the IVW method. The MR-Egger regression method needed to satisfy the InSIDE (instrument strength independent of direct effect assumption, which weakens the exclusivity assumption in the IVW method. The WM assumed that there is pleiotropy in less than 50% of SNPs and the median of the distribution function obtained by ordering the effect sizes of all individual SNPs by weight.
		d)	Explain how missing data were addressed		Not applicable
		e)	If applicable, indicate how multiple testing was addressed		Not applicable
7	**Assessment of assumptions**		Describe any methods or prior knowledge used to assess the assumptions or justify their validity	6	TSMR Analysis was performed in this experiment using five main methods: inverse variance weighting (IVW), MR-Egger regression, weighted median (WM), simple mode, and weighted mode. The primary analysis method was IVW to evaluate the presupposed SNPs as valid IVs and the ratio method was used to calculate the single SNP to obtain the estimate under the premise of no horizontal pleiotropy, thereby precisely estimating the aggregate causal effect between exposure and outcome. MR-Egger regression and the WM were complementary to the IVW method. The MR-Egger regression method needed to satisfy the InSIDE (instrument strength independent of direct effect assumption, which weakens the exclusivity assumption in the IVW method. The WM assumed that there is pleiotropy in less than 50% of SNPs and the median of the distribution function obtained by ordering the effect sizes of all individual SNPs by weight.

8	**Sensitivity analyses and additional analyses**		Describe any sensitivity analyses or additional analyses performed (e.g. comparison of effect estimates from different approaches, independent replication, bias analytic techniques, validation of instruments, simulations)	7	Statistical heterogeneity among SNPs was assessed by Cochran Q test, and P < 0.05 was considered statistically significant. MR-Egger intercept term was used to test whether there was gene pleiotropy between circulating inflammatory factors and MG. If the intercept was different from zero, it indicated that there was horizontal pleiotropy in the study, otherwise there was not exist. Leave-one-out analysis was used to gradually remove SNPs, calculate the combined effect of the remaining SNPs and observe the influence of each SNP on the results to determine the degree of SNP influence on the results. All methods used in this study were analyzed using the TwoSampleMR package in R (Version 4.3.0) .
9	**Software and pre-registration**				
		a)	Name statistical software and package(s), including version and settings used	7	All methods used in this study were analyzed using the TwoSampleMR package in R (Version 4.3.0)./ BWMR was performed using 'BWMR' package in R software (Version 4.3.0).
		b)	State whether the study protocol and details were pre-registered (as well as when and where)	6	This study protocol and details are not pre-registered.
	**RESULTS**				
10	**Descriptive data**				
		a)	Report the numbers of individuals at each stage of included studies and reasons for exclusion. Consider use of a flow diagram	6	We set P<5×10-8 as the genome-wide significant threshold to select strongly associated SNPs with MG and inflammatory cytokines. We found only 4 SNPs for cytokines at P<5×10−8. Therefore, a significance threshold (P<5×10−6) was used to select instrumental variables, which could better represent the genetic variation of exposure factors and effectively evaluate the association between exposure and outcome in the subsequent inference process. (Supplementary Table 1、2). To remove linkage disequilibrium, we set the screening condition (r2=0.001, kb=10000) to ensure that the choice of IVs was independent of each other. Finally, the weak instrumental variables were removed using F-statistic＞10 as the criterion

				(Supplementary Table 1、2)./ Detailed values are provided in the Supplementary table.
	b)	Report summary statistics for phenotypic exposure(s), outcome(s), and other relevant variables (e.g. means, SDs, proportions)	9	For each 1-standard deviation (SD) increase in ADA (OR=1.16, 95%CI: 1.00-1.33; P=0.041) and CD40L receptor (OR=1.20, 95%CI: 1.02-1.40; P=0.025) levels, the risk of MG increased by 16% and 20%, respectively. In addition, IL-1α (OR=0.74, 95%CI:0.58~0.0.96;P=0.022)、GDNF (OR=0.76, 95% CI: 0.61 ~ 0.94; P=0.013)、OPG (OR=0.76, 95% CI: 0.61 ~ 0.94; P=0.012) and TNF-β (OR=0.80, 95% CI: 0.68 ~ 0.93; P=0.006) were negatively associated with the risk of MG. MR-Egger analysis showed that higher IL-1α was associated with the lower risk of MG, and for each 1-SD increase, the risk of MG as reduced by 31% (OR=0.61; 95%CI: 0.39-0.96; P=0.045). The funnel plots are shown in Supplementary figure 3. We used the BWMR method to validate our findings, which demonstrated that the above six inflammatory cytokines were associated with a high risk of MG (Figure 2、Supplementary table 9).
	c)	If the data sources include meta-analyses of previous studies, provide the assessments of heterogeneity across these studies		Not involved
	d)	For two-sample MR:<br>i. Provide justification of the similarity of the genetic variant-exposure associations between the exposure and outcome samples<br>ii. Provide information on the number of individuals who overlap between the exposure and outcome studies	8	We set P<5×10-8 as the genome-wide significant threshold to select strongly associated SNPs with MG and inflammatory cytokines. We found only 4 SNPs for cytokines at P<5×10−8. Therefore, a significance threshold (P<5×10−6) was used to select instrumental variables, which could better represent the genetic variation of exposure factors and effectively evaluate the association between exposure and outcome in the subsequent inference process. (Supplementary Table 1、2). To remove linkage disequilibrium, we set the screening condition (r2=0.001, kb=10000) to ensure that the choice of IVs was independent of each other. Finally, the weak instrumental variables were removed using F-statistic＞10 as the criterion (Supplementary Table 1、2)/Prior to MR Analysis, we preprocessed the data to ensure that there was no overlap between exposure GWAS data and outcome GWAS data. We removed duplicate SNPS by methods such as Clumping, ensuring that

				SNPS in each sample were unique./ Detailed values are provided in the Supplementary table.
11	**Main results**			
	a)	Report the associations between genetic variant and exposure, and between genetic variant and outcome, preferably on an interpretable scale	8、11	The effect of 91 circulating inflammatory cytokines on the risk of MG is shown in the forest plot (Figure 2). The results of IVW showed that seven inflammatory cytokines were associated with the risk of MG, including adenosine deaminase (ADA)、CD40L receptor、C-X-C motif chemokine 11 (CXCL11)、interleukin-1-alpha (IL-1α)、glial-cell-line-derived neurotrophic factor (GDNF)、Osteoprotegerin (OPG) and tumor necrosis factor-beta (TNF-β) (Table 3、Figure 3). Genetically predicted ADA and CD40L were positively correlated with the risk of MG, indicating that the levels of these two inflammatory cytokines have adverse effects on MG. For each 1-standard deviation (SD) increase in ADA (OR=1.16, 95%CI: 1.00-1.33; P=0.041) and CD40L receptor (OR=1.20, 95%CI: 1.02-1.40; P=0.025) levels, the risk of MG increased by 16% and 20%, respectively. In addition, IL-1α (OR=0.74, 95%CI:0.58~0.0.96;P=0.022)、GDNF (OR=0.76, 95% CI: 0.61 ~ 0.94; P=0.013)、OPG (OR=0.76, 95% CI: 0.61 ~ 0.94; P=0.012) and TNF-β (OR=0.80, 95% CI: 0.68 ~ 0.93; P=0.006) were negatively associated with the risk of MG. MR-Egger analysis showed that higher IL-1α was associated with the lower risk of MG, and for each 1-SD increase, the risk of MG as reduced by 31% (OR=0.61; 95%CI: 0.39-0.96; P=0.045). The funnel plots are shown in Supplementary figure 3. We used the BWMR method to validate our findings, which demonstrated that the above six inflammatory cytokines were associated with a high risk of MG (Figure 2、Supplementary table 9).<br><br>The results of reverse MR analysis of MG and 91 circulating inflammatory cytokines are shown in the forest plot (Figure 5、Supplementary Table 4). The results of IVW showed that IL-1α (OR=0.96; 95%CI: 0.94-0.99; P=0.006) was negatively associated with MG risk in the reverse MR analysis. Furthermore, the risk of MG was found to be positively associated with C-C motif chemokine

			19 (CCL19) (OR=1.05, 95%CI:1.02~1.07; P < 0.001)、TNF-related activation-induced cytokine (TRANCE) (OR=1.04, 95%CI:1.02~1.07;P=0.006)、TNF-β (OR=1.03, 95%CI:1.00~1.06;P=0.017)、macrophage inflammatory protein 1a (MIP-1α) (OR=1.02, 95%CI:1.00~1.05;P=0.04)、interleukin-12 subunit beta (IL-12β) (OR=1.04, 95%CI:1.01~1.07;P=0.002), and negatively associated with Delta and Notch-like epidermal growth factor-related receptor (DNER)(OR=0.97, 95%CI:0.95~1.00; P=0.02) and IL-1α (OR=0.96, 95%CI:0.94~0.99;P=0.006) (Supplementary figure 5). The funnel plots of inflammatory cytokines are shown in Supplementary figure 4. We used the BWMR method to validate our findings, which demonstrated that the above seven inflammatory cytokines were associated with a high risk of MG (Figure 4、Supplementary table 10).
	b) Report MR estimates of the relationship between exposure and outcome, and the measures of uncertainty from the MR analysis, on an interpretable scale, such as odds ratio or relative risk per SD difference	8、11	The effect of 91 circulating inflammatory cytokines on the risk of MG is shown in the forest plot (Figure 2). The results of IVW showed that seven inflammatory cytokines were associated with the risk of MG, including adenosine deaminase (ADA)、CD40L receptor、C-X-C motif chemokine 11 (CXCL11)、interleukin-1-alpha (IL-1α)、glial-cell-line-derived neurotrophic factor (GDNF)、Osteoprotegerin (OPG) and tumor necrosis factor-beta (TNF-β) (Table 3、Figure 3). Genetically predicted ADA and CD40L were positively correlated with the risk of MG, indicating that the levels of these two inflammatory cytokines have adverse effects on MG. For each 1-standard deviation (SD) increase in ADA (OR=1.16, 95%CI: 1.00-1.33; P=0.041) and CD40L receptor (OR=1.20, 95%CI: 1.02-1.40; P=0.025) levels, the risk of MG increased by 16% and 20%, respectively. In addition, IL-1α (OR=0.74, 95%CI:0.58~0.0.96;P=0.022)、GDNF (OR=0.76, 95% CI: 0.61 ~ 0.94; P=0.013)、OPG (OR=0.76, 95% CI: 0.61 ~ 0.94; P=0.012) and TNF-β (OR=0.80, 95% CI: 0.68 ~ 0.93; P=0.006) were negatively associated with the risk of MG. MR-Egger analysis showed that higher IL-1α was associated with the lower risk of MG, and for each

				1-SD increase, the risk of MG as reduced by 31% (OR=0.61; 95%CI: 0.39-0.96; P=0.045). The funnel plots are shown in Supplementary figure 3. We used the BWMR method to validate our findings, which demonstrated that the above six inflammatory cytokines were associated with a high risk of MG (Figure 2、Supplementary table 9).<br><br>The results of reverse MR analysis of MG and 91 circulating inflammatory cytokines are shown in the forest plot (Figure 5、Supplementary Table 4). The results of IVW showed that IL-1α (OR=0.96; 95%CI: 0.94-0.99; P=0.006) was negatively associated with MG risk in the reverse MR analysis. Furthermore, the risk of MG was found to be positively associated with C-C motif chemokine 19 (CCL19) (OR=1.05, 95%CI:1.02~1.07; P＜0.001)、TNF-related activation-induced cytokine (TRANCE) (OR=1.04, 95%CI:1.02~1.07;P=0.006)、TNF-β (OR=1.03, 95%CI:1.00~1.06;P=0.017)、macrophage inflammatory protein 1a (MIP-1α) (OR=1.02, 95%CI:1.00~1.05;P=0.04)、interleukin-12 subunit beta (IL-12β) (OR=1.04, 95%CI:1.01~1.07;P=0.002), and negatively associated with Delta and Notch-like epidermal growth factor-related receptor (DNER)(OR=0.97, 95%CI:0.95~1.00; P=0.02) and IL-1α (OR=0.96, 95%CI:0.94~0.99;P=0.006) (Supplementary figure 5). The funnel plots of inflammatory cytokines are shown in Supplementary figure 4. We used the BWMR method to validate our findings, which demonstrated that the above seven inflammatory cytokines were associated with a high risk of MG (Figure 4、Supplementary table 10).
	c)	If relevant, consider translating estimates of relative risk into absolute risk for a meaningful time period	8	For each 1-standard deviation (SD) increase in ADA (OR=1.16, 95%CI: 1.00-1.33; P=0.041) and CD40L receptor (OR=1.20, 95%CI: 1.02-1.40; P=0.025) levels, the risk of MG increased by 16% and 20%, respectively./ MR-Egger analysis showed that higher IL-1α was associated with the lower risk of MG, and for each 1-SD increase, the risk of MG as reduced by 31% (OR=0.61; 95%CI: 0.39-0.96; P=0.045).

		d)	Consider plots to visualize results (e.g. forest plot, scatterplot of associations between genetic variants and outcome versus between genetic variants and exposure)	9、10、12	The results of MR analysis of 91 circulating inflammatory cytokines and MG risk are shown in the forest plot (Figure 2). / There was no significant difference in the MR-Egger analysis of the other five inflammatory factors (Figure 3). The funnel plots of cytokines are shown in Figure 4.
12	**Assessment of assumptions**				
		a)	Report the assessment of the validity of the assumptions	6	Supplementary Table1、2
		b)	Report any additional statistics (e.g., assessments of heterogeneity across genetic variants, such as I^2, Q statistic or E-value)	16	The MR-Egger intercept test and the Cochran Q test found no significant horizontal pleiotropy or heterogeneity among these inflammatory cytokines (Supplementary tables 5-8). Leave-one-out analysis indicated that no single SNP significantly affected the results of MR (Supplementary figure 1 and 2).
13	**Sensitivity analyses and additional analyses**				
		a)	Report any sensitivity analyses to assess the robustness of the main results to violations of the assumptions	8-12	Supplementary tables 5-8
		b)	Report results from other sensitivity analyses or additional analyses	8-12	Supplementary tables 5-8
		c)	Report any assessment of direction of causal relationship (e.g., bidirectional MR)	8-12	Supplementary tables 4-5
		d)	When relevant, report and compare with estimates from non-MR analyses		Not mentioned.
		e)	Consider additional plots to visualize results (e.g., leave-one-out analyses)	11	Leave-one-out analysis indicated that no single SNP significantly affected the results of MR (Supplementary figure 1 and 2).
	**DISCUSSION**				
14	**Key results**		Summarize key results with reference to study objectives	17	The results showed that ADA and CD40L were positively associated with the risk of MG, and CXCL11、IL-1α、GDNF、OPG and TNF-β were negatively associated with the risk of MG. Inverse MR analysis showed that the risk of MG was associated with CCL19、TRANCE、IL-12β、TNF-β、MIP-1α、IL-1β and DNER when MG genetic data were used as exposure factors. There is a

					bidirectional causal relationship between IL-1α and TNF-β and the risk of MG, which may be located in the upstream and downstream of MG progression. Inflammatory cytokines with bidirectional causal association may be important markers in the progression of MG disease.
15	**Limitations**		Discuss limitations of the study, taking into account the validity of the IV assumptions, other sources of potential bias, and imprecision. Discuss both direction and magnitude of any potential bias and any efforts to address them	18	Our study has some limitations. Firstly, since all MG patients were from Italy and the United States, this study may not be available in other ethnic groups, so further exploration of applicability to other ethnic groups is required. Secondly, we have lowered the threshold standard for screening IVs, but we still could not be completely excluded the influence of weak instrument effect on the outcome. In the future, researchers need to expand the sample size of MG and include different ethnic groups for research. Thirdly, we considered that AchR MG is a heterogeneous population and that the severity and course of the disease as well as the use of immunosuppressive agents may alter the levels of inflammatory cytokines. A large number of observational studies are needed to confirm our results.
16	**Interpretation**				
		a)	Meaning: Give a cautious overall interpretation of results in the context of their limitations and in comparison, with other studies	19	Our study found that IL-1α was negatively associated with the risk of MG, which was contrary to the results of previous observational studies, but the results of the simple mode algorithm showed that IL-1α was negatively associated with the risk of MG. We believe that it is due to the difference in results brought by different algorithms, but the specific mechanism is not clear. / The inverse association of TNF-β with the risk of MG in our study contradicts previous findings, and we suggested that it may be due to confounding bias in observational experiments or different genetic associations among different populations.
		b)	Mechanism: Discuss underlying biological mechanisms that could drive a potential causal relationship between the investigated exposure and the outcome, and whether the gene-environment equivalence assumption is reasonable. Use causal language carefully, clarifying that IV estimates may provide causal effects only under certain assumptions	21	We have lowered the threshold standard for screening IV and used MR Steiger method to screen IVs, but we still could not be completely excluded the influence of weak instrument effect on the outcome. In the future, researchers need to expand the sample size of MG and include different ethnic groups for research. More importantly, there

				may be differences in genetic associations between populations in GWAS of circulating inflammatory factors and in GWAS of MG.
		c) Clinical relevance: Discuss whether the results have clinical or public policy relevance, and to what extent they inform effect sizes of possible interventions	21	Overall, the results of our MR analysis were not similar to those of some previous clinical trials, which we analysed may be related to the different stages of the disease or various different factors in life activities.
17	**Generalizability**	Discuss the generalizability of the study results (a) to other populations, (b) across other exposure periods/timings, and (c) across other levels of exposure	18	Firstly, since all MG patients were from Italy and the United States, this study may not be available in other ethnic groups, so further exploration of applicability to other ethnic groups is required.
	**OTHER INFORMATION**			
18	**Funding**	Describe sources of funding and the role of funders in the present study and, if applicable, sources of funding for the databases and original study or studies on which the present study is based	22	This research did not receive from funding agencies in the public, commercial, or not-for-profit sectors.
19	**Data and data sharing**	Provide the data used to perform all analyses or report where and how the data can be accessed, and reference these sources in the article. Provide the statistical code needed to reproduce the results in the article, or report whether the code is publicly accessible and if so, where	22	The original contributions presented in the study are included in the Supplementary Material and can be directed to the corresponding author if further detailed inquiries are required.
20	**Conflicts of Interest**	All authors should declare all potential conflicts of interest	22	The authors declare that the research was conducted in the absence of any commercial or financial relationships that could be construed as a potential conflict of interest.

This checklist is copyrighted by the Equator Network under the Creative Commons Attribution 3.0 Unported (CC BY 3.0) license.

1. Skrivankova VW, Richmond RC, Woolf BAR, Yarmolinsky J, Davies NM, Swanson SA, et al. Strengthening the Reporting of Observational Studies in Epidemiology using Mendelian Randomization (STROBE-MR) Statement. JAMA. 2021;under review.

2. Skrivankova VW, Richmond RC, Woolf BAR, Davies NM, Swanson SA, VanderWeele TJ, et al. Strengthening the Reporting of Observational Studies in Epidemiology using Mendelian Randomisation (STROBE-MR): Explanation and Elaboration. BMJ. 2021;375:n2233.
