# Supplementary material for: Circulating inflammatory cytokines and the risk of myasthenia gravis: a bidirectional Mendelian randomization study
Source: BMC Neurol. 2025 Jul 1;25:271. doi: 10.1186/s12883-025-04271-9 (PMC12211973; doi:10.1186/s12883-025-04271-9)
Supplement: Supplementary file 3 — Supplementary Material 3 [file 12883_2025_4271_MOESM3_ESM.pdf]

Sequence of pictures:ADA,CD40L,GDNF
,IL-1α,OPG,TNF-β

The horizontal axis represents the strength of the association between the instrumental variable and the exposure.The vertical axis represents the correlation strength between the instrumental variable and the outcome. Each point corresponds to an instrumental variable used for analysis.

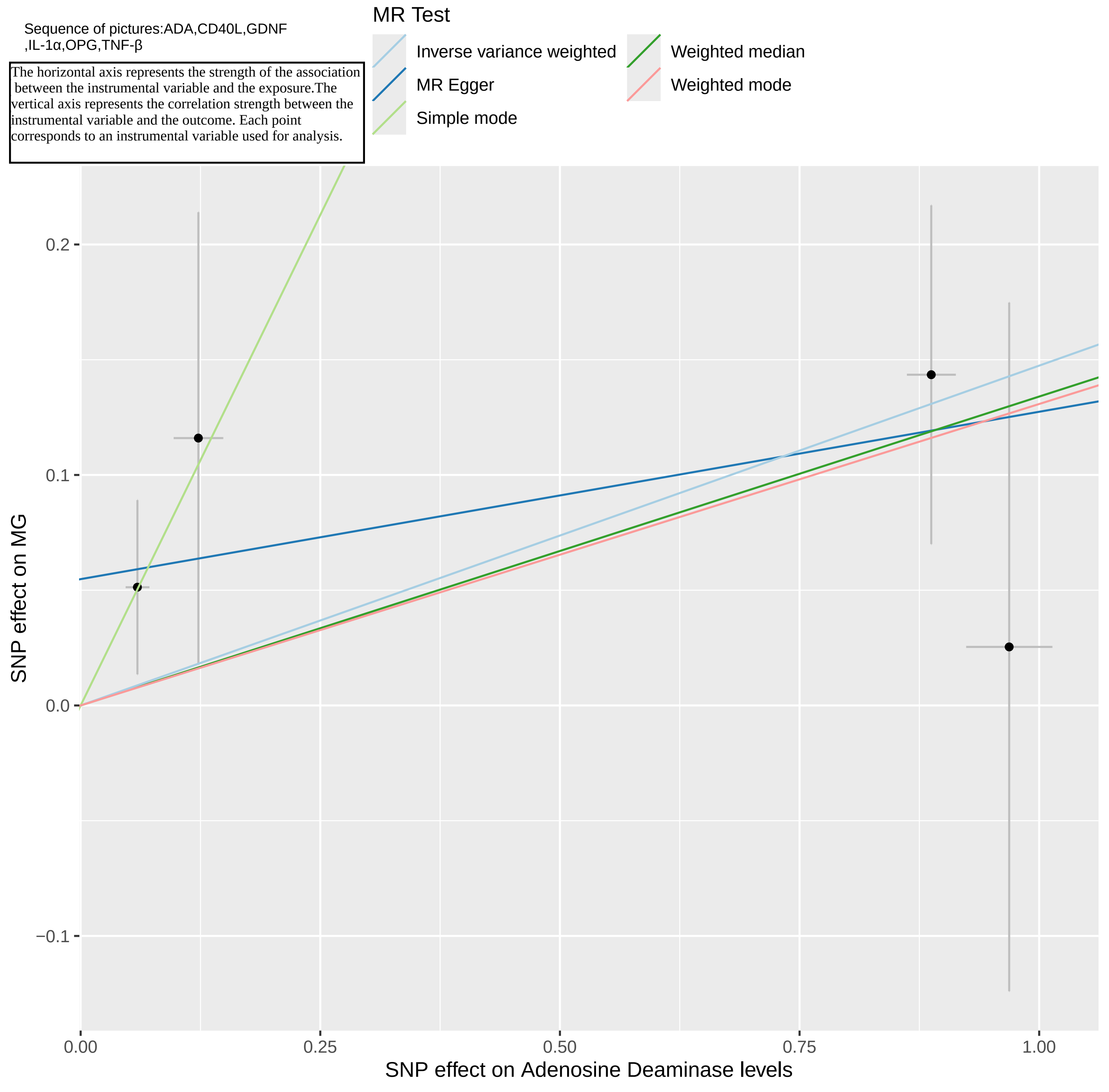

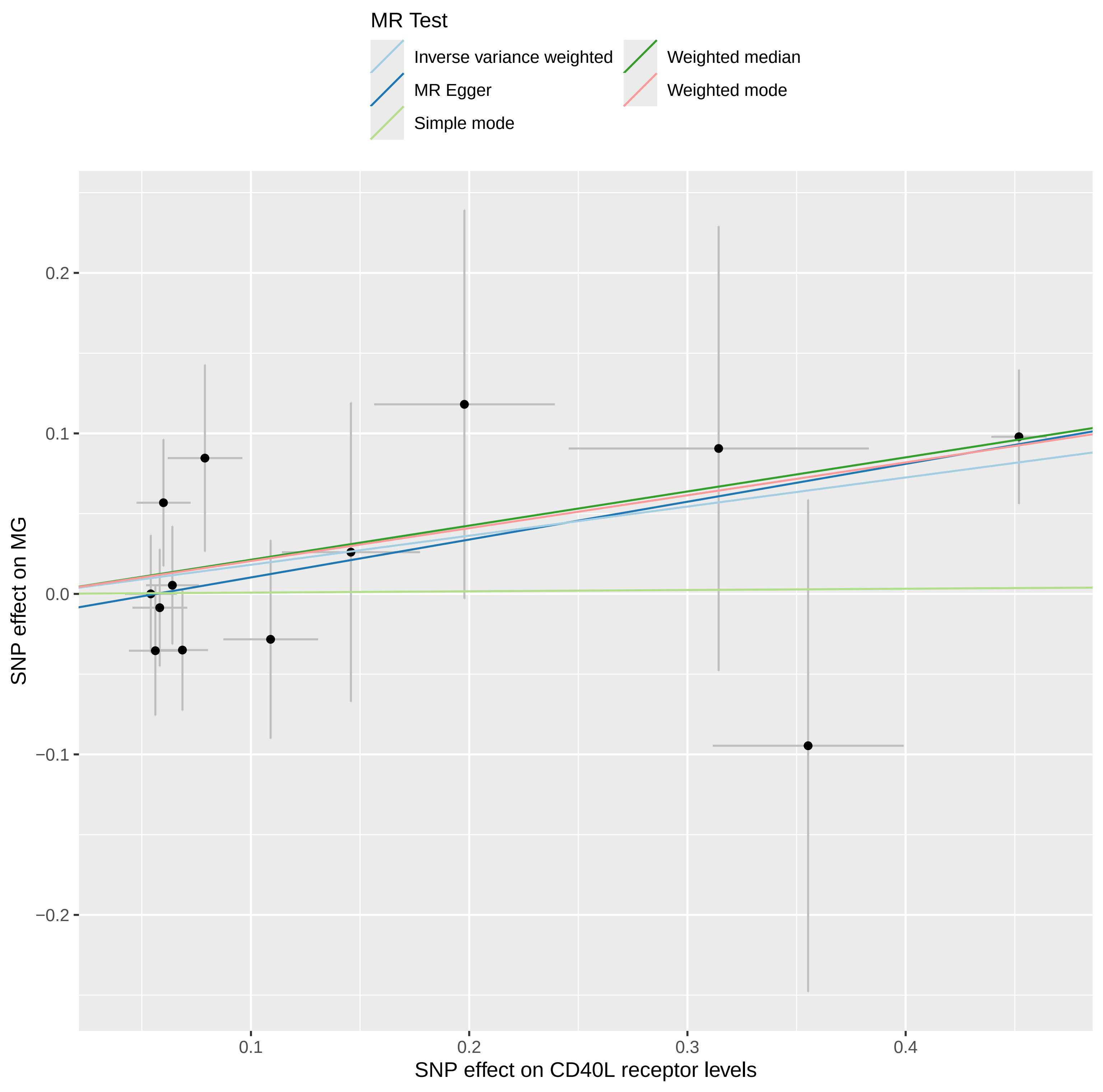

MR Test
Inverse variance weighted
MR Egger
Simple mode
Weighted median
Weighted mode
SNP effect on MG
SNP effect on CD40L receptor levels
0.2
0.1
0.0
−0.1
−0.2
0.1
0.2
0.3
0.4

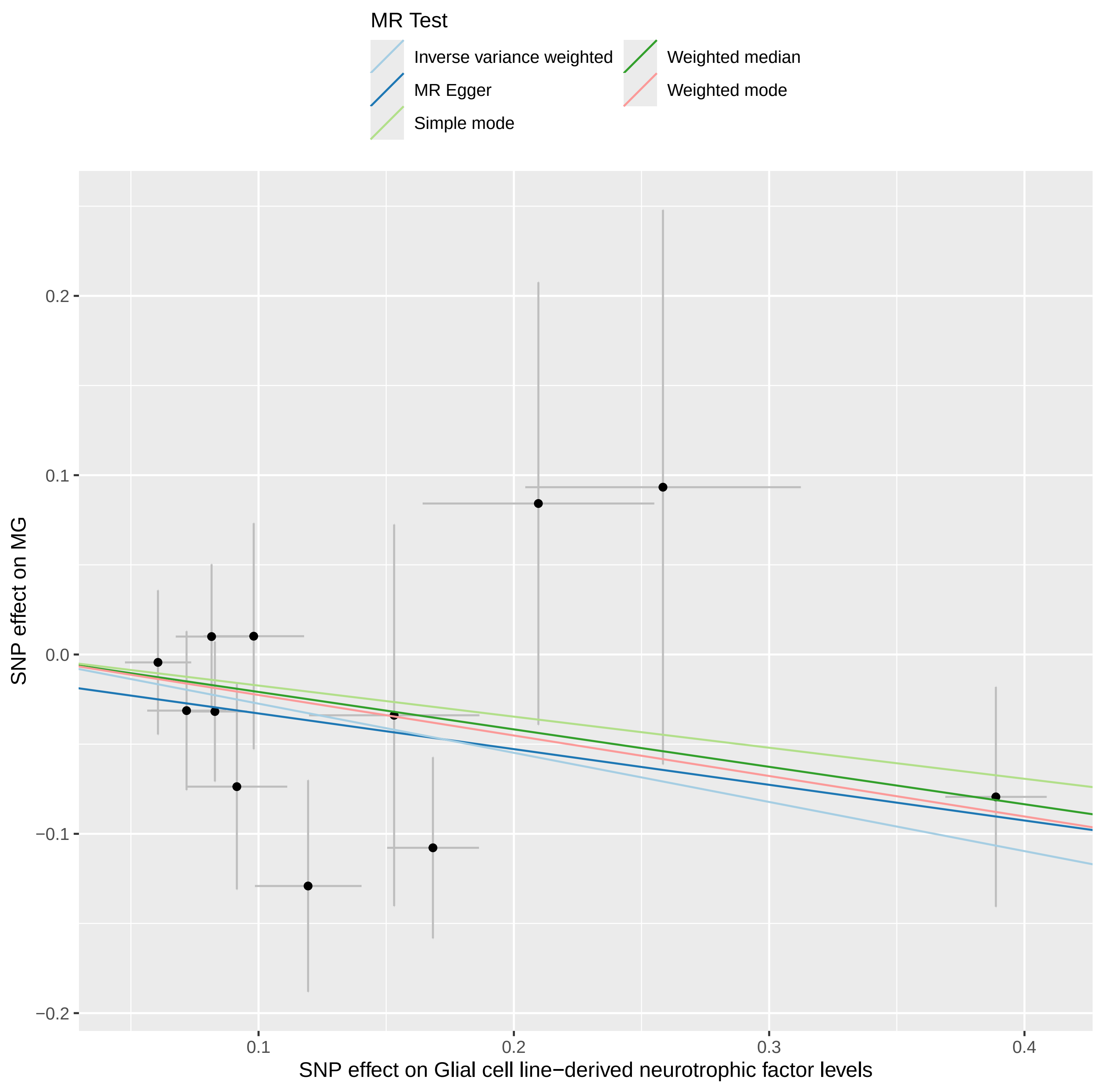
MR Test
Inverse variance weighted
MR Egger
Simple mode
Weighted median
Weighted mode
0.2
0.1
0.0
−0.1
−0.2
SNP effect on MG
0.1
0.2
0.3
0.4
SNP effect on Glial cell line−derived neurotrophic factor levels

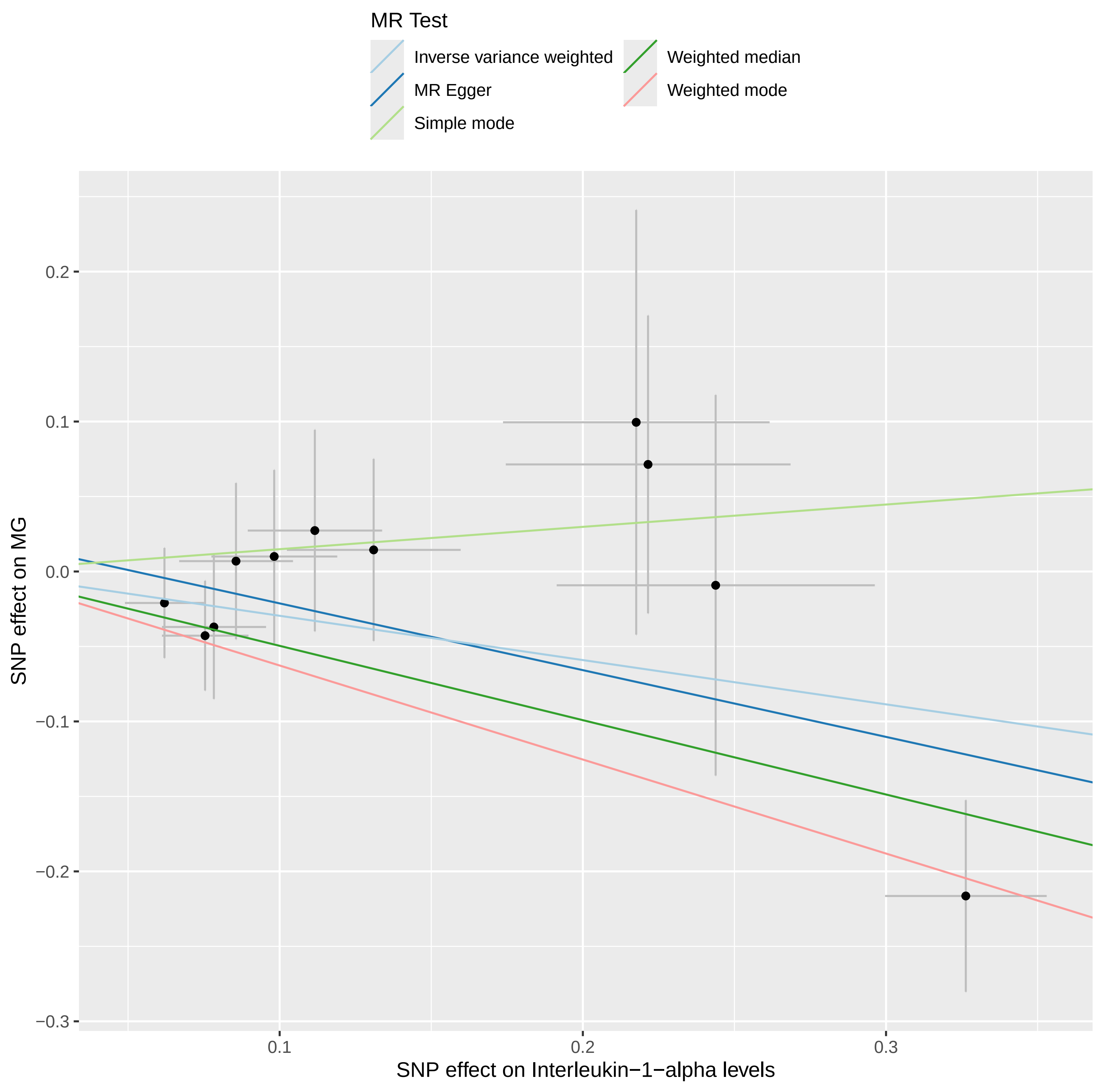
MR Test
Inverse variance weighted
MR Egger
Simple mode
Weighted median
Weighted mode
SNP effect on MG
0.2
0.1
0.0
−0.1
−0.2
−0.3
0.1
0.2
0.3
SNP effect on Interleukin−1−alpha levels

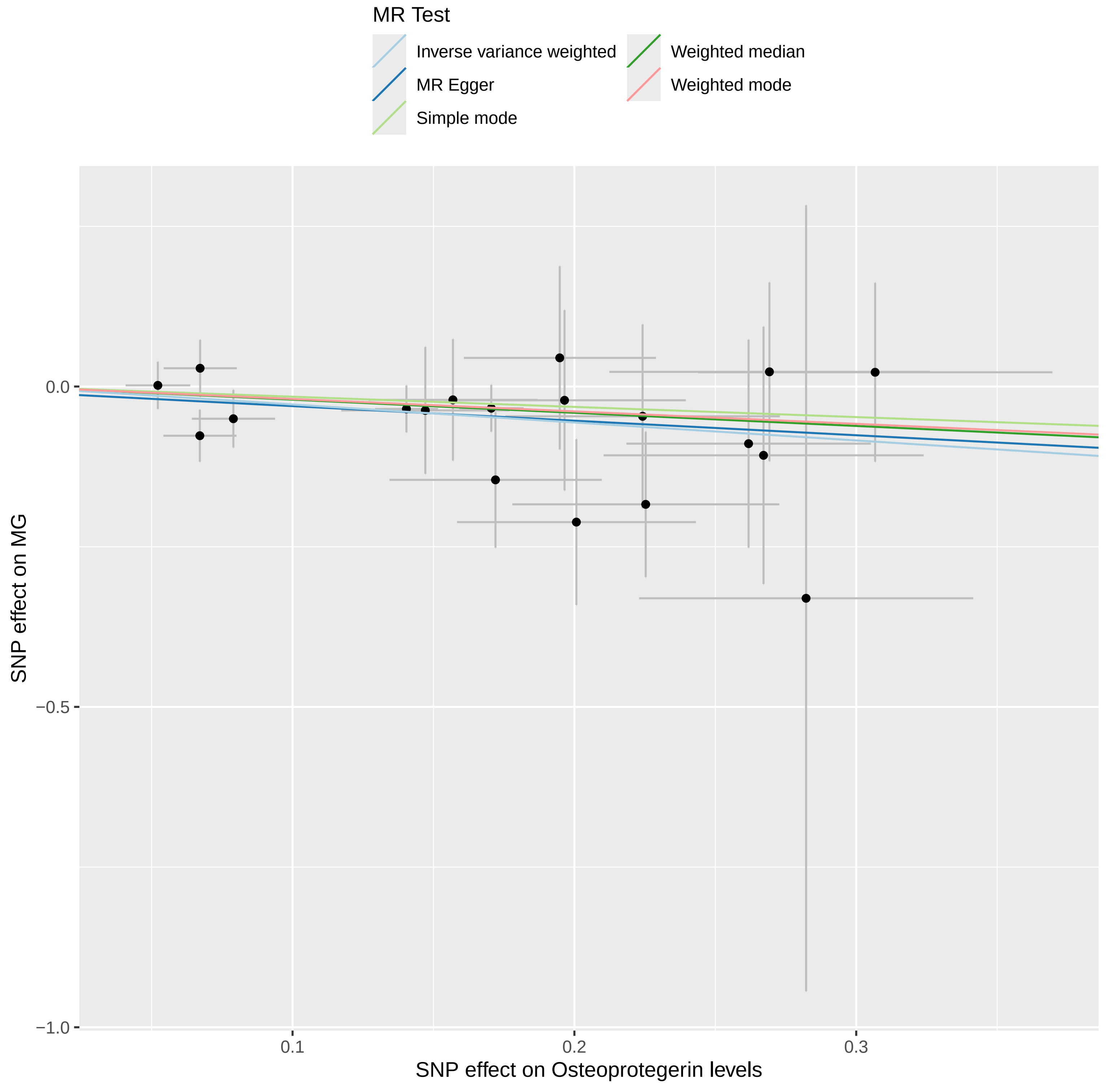
MR Test
Inverse variance weighted
MR Egger
Simple mode
Weighted median
Weighted mode
SNP effect on MG
0.0
−0.5
−1.0
0.1
0.2
0.3
SNP effect on Osteoprotegerin levels

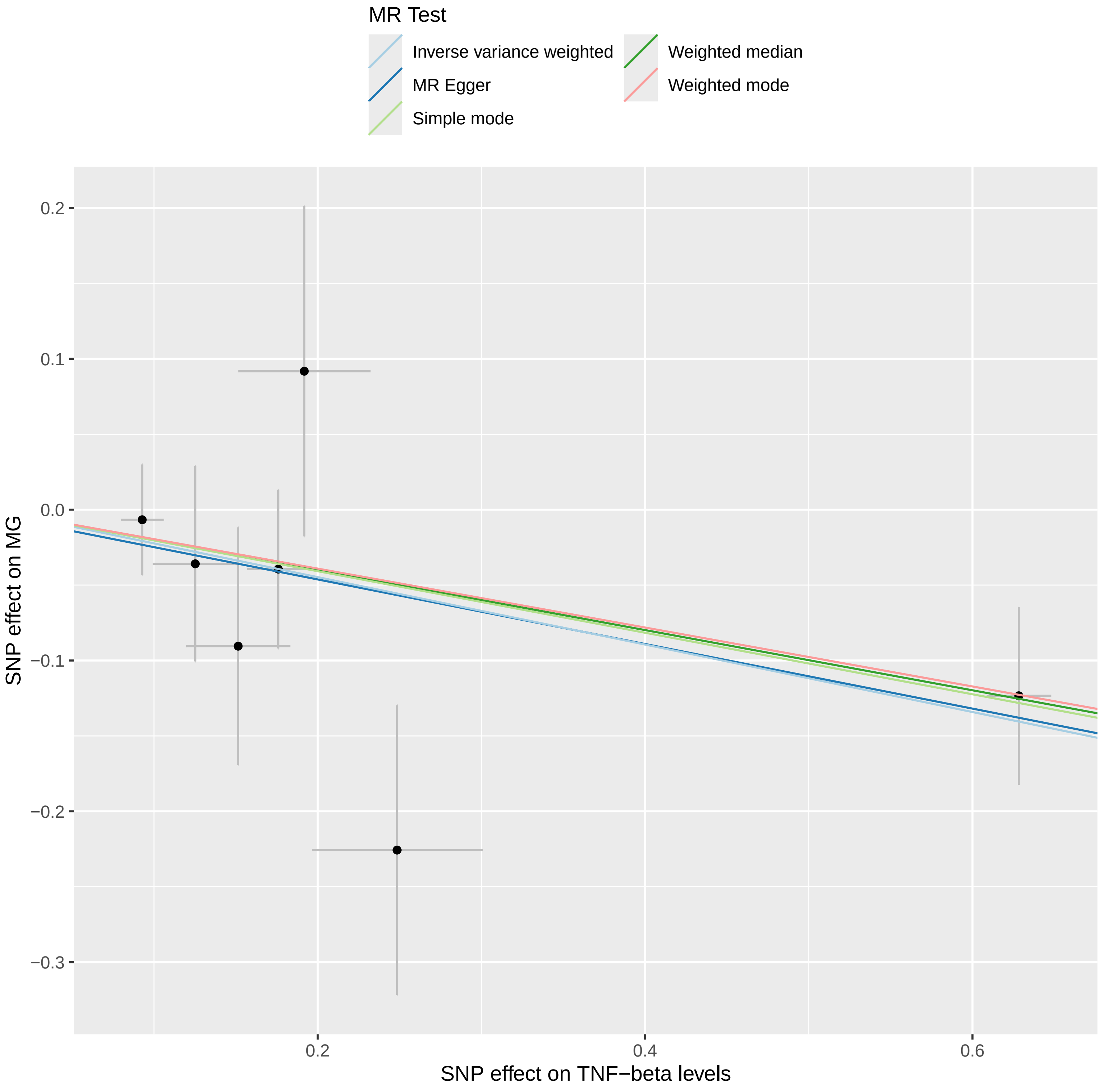

MR Test
Inverse variance weighted
MR Egger
Simple mode
Weighted median
Weighted mode
0.2
0.1
0.0
−0.1
−0.2
−0.3
SNP effect on MG
0.2
0.4
0.6
SNP effect on TNF−beta levels
