# Supplementary material for: Circulating inflammatory cytokines and the risk of myasthenia gravis: a bidirectional Mendelian randomization study
Source: BMC Neurol. 2025 Jul 1;25:271. doi: 10.1186/s12883-025-04271-9 (PMC12211973; doi:10.1186/s12883-025-04271-9)
Supplement: Supplementary file 4 — Supplementary Material 4 [file 12883_2025_4271_MOESM4_ESM.pdf]

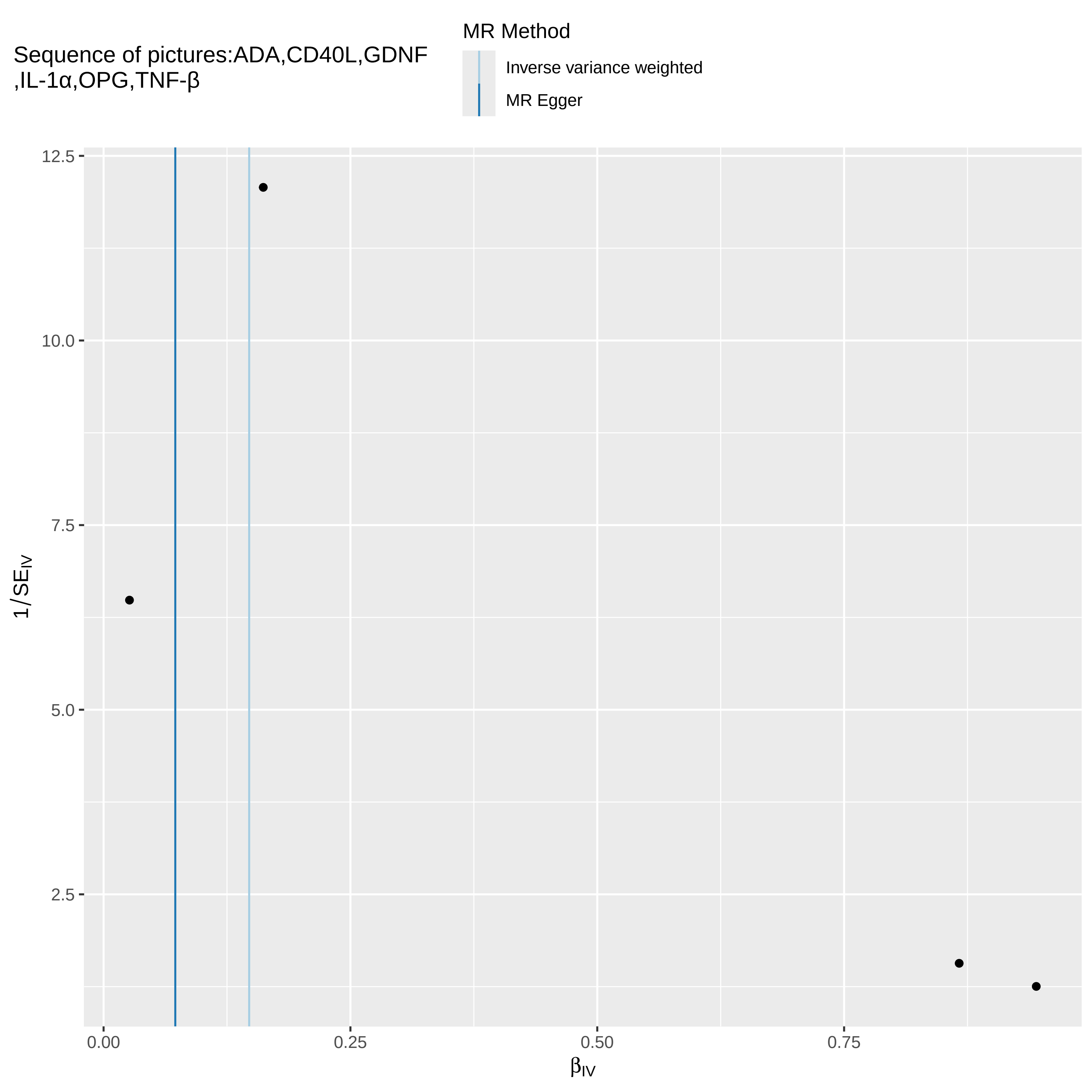
Sequence of pictures:ADA,CD40L,GDNF
,IL-1α,OPG,TNF-β
MR Method
Inverse variance weighted
MR Egger
12.5
10.0
7.5
5.0
2.5
0.00
0.25
0.50
0.75
1/SE_IV
β_IV

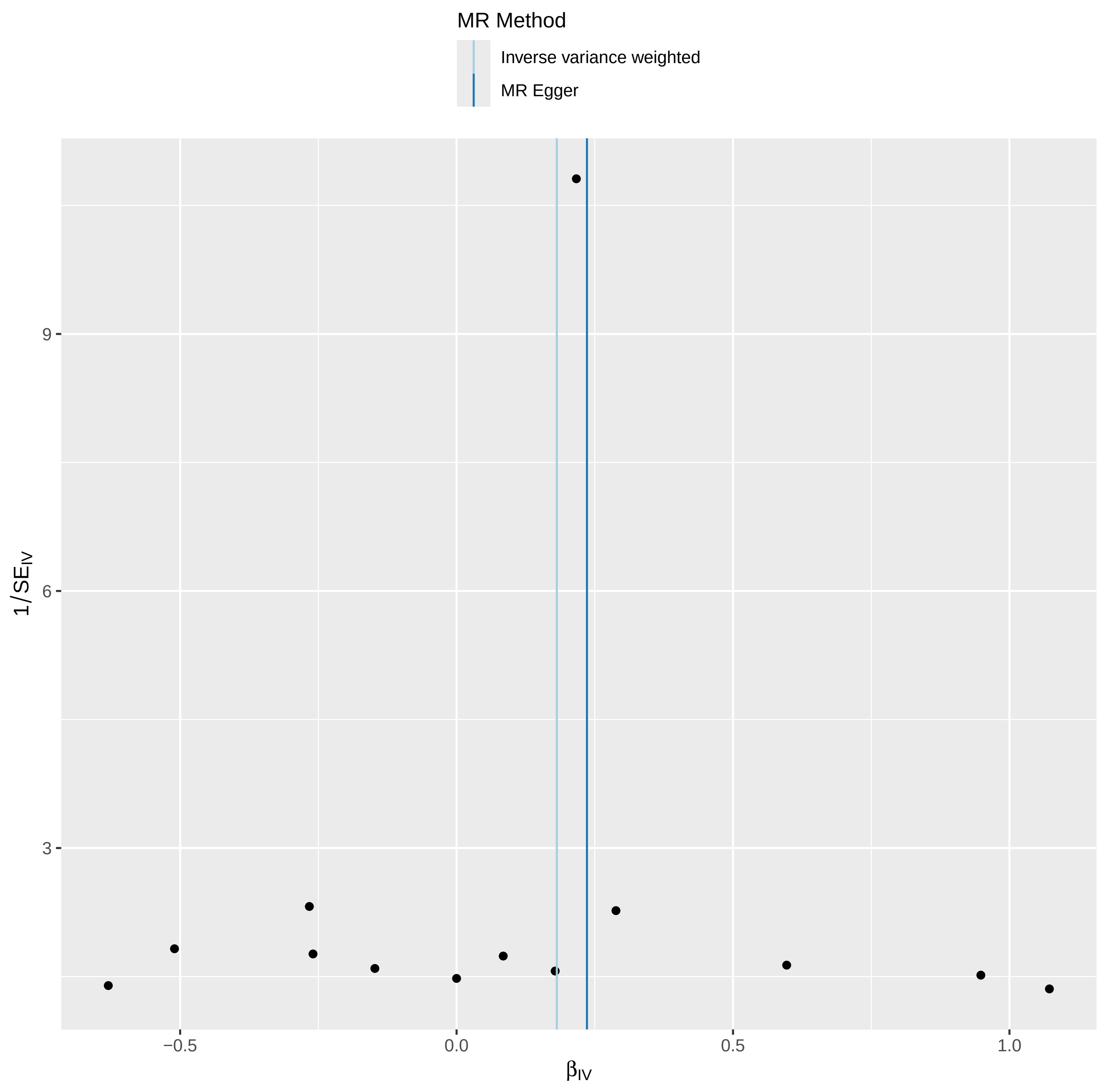

MR Method
Inverse variance weighted
MR Egger
9
6
3
$1/SE_{IV}$
−0.5
0.0
0.5
1.0
β_{IV}

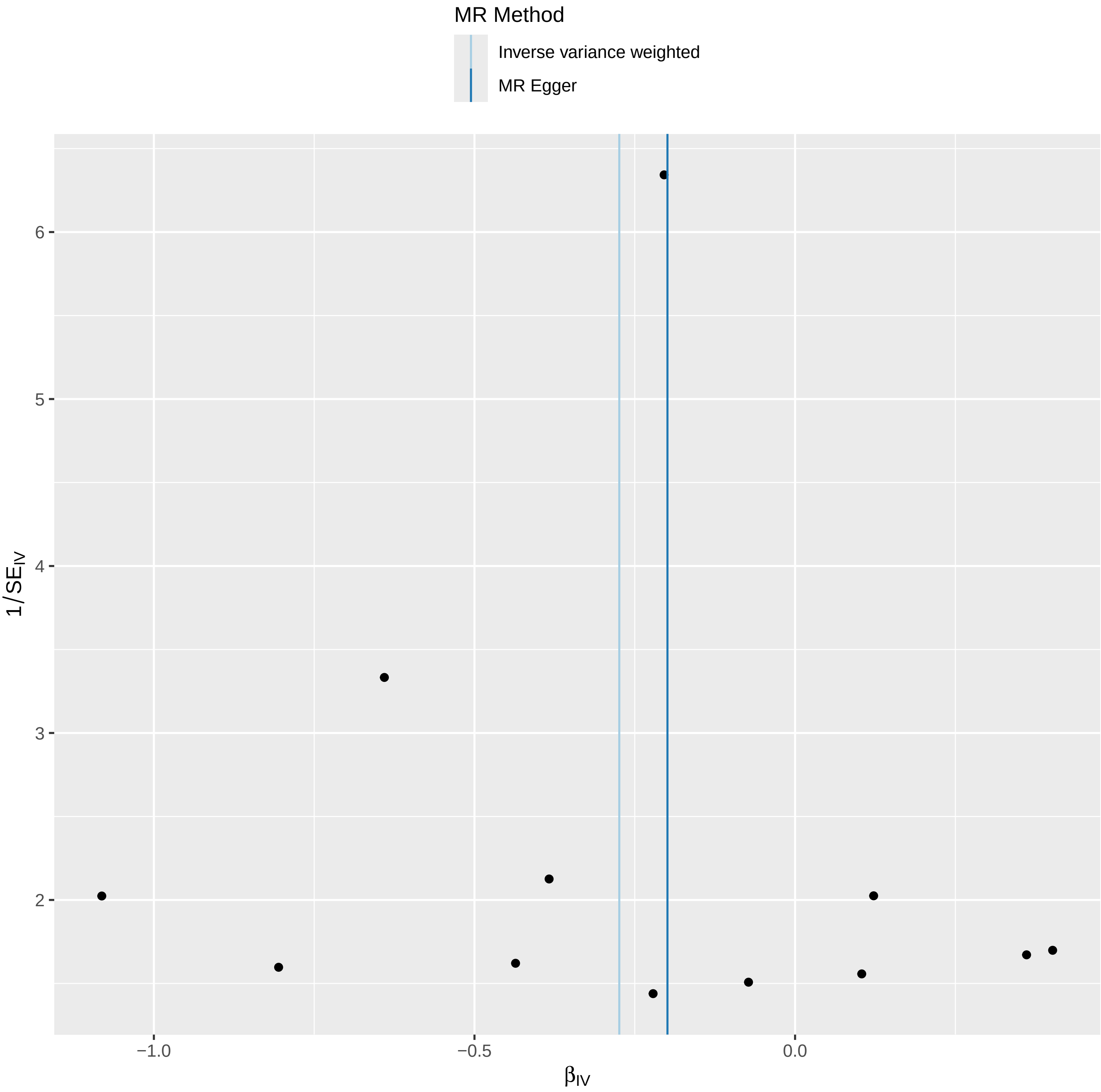

MR Method
Inverse variance weighted
MR Egger
6
5
4
3
2
1/SE_IV
−1.0
−0.5
0.0
β_IV

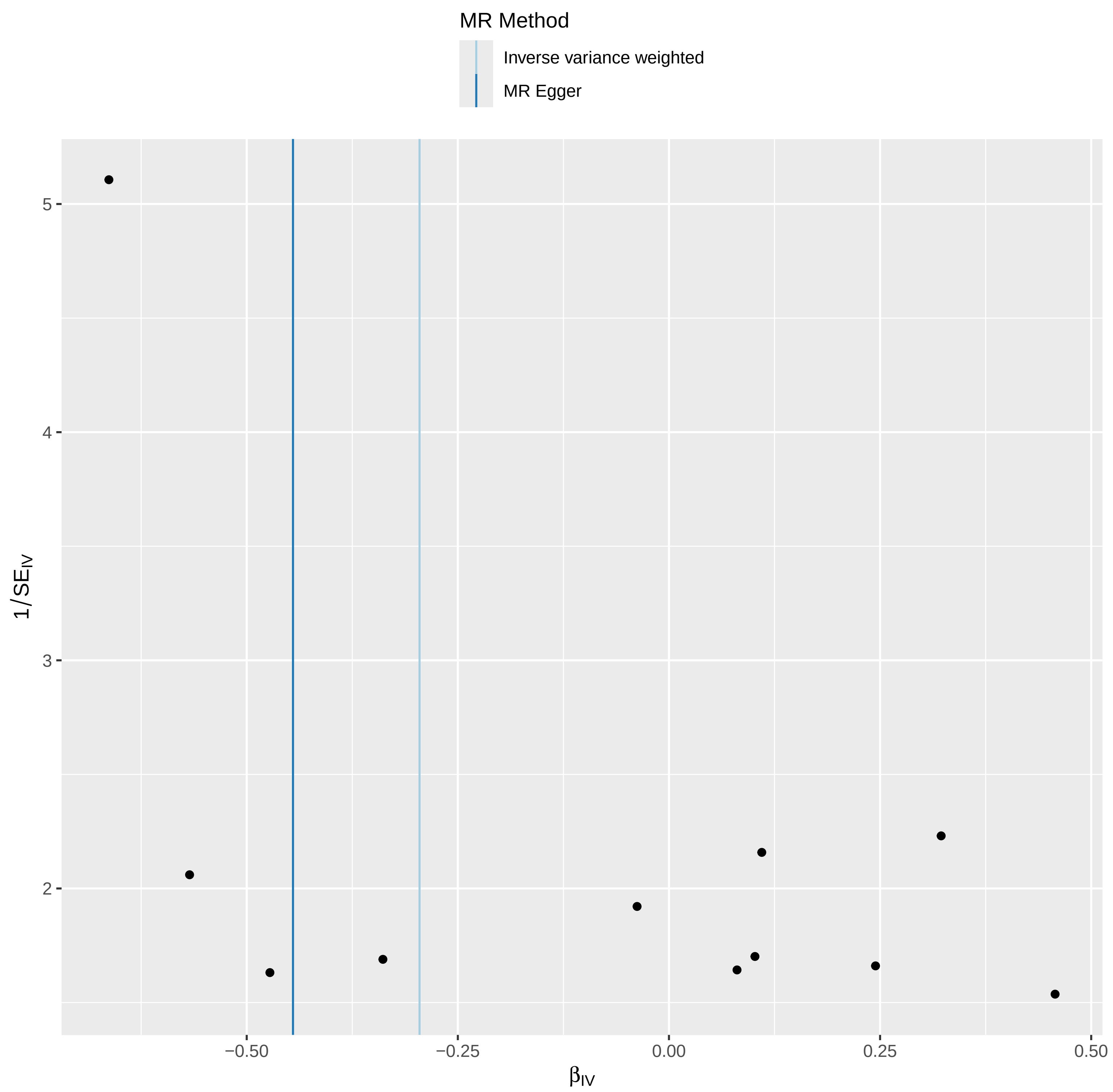
MR Method
Inverse variance weighted
MR Egger
5
4
3
2
1/SE_IV
−0.50
−0.25
0.00
0.25
0.50
β_IV

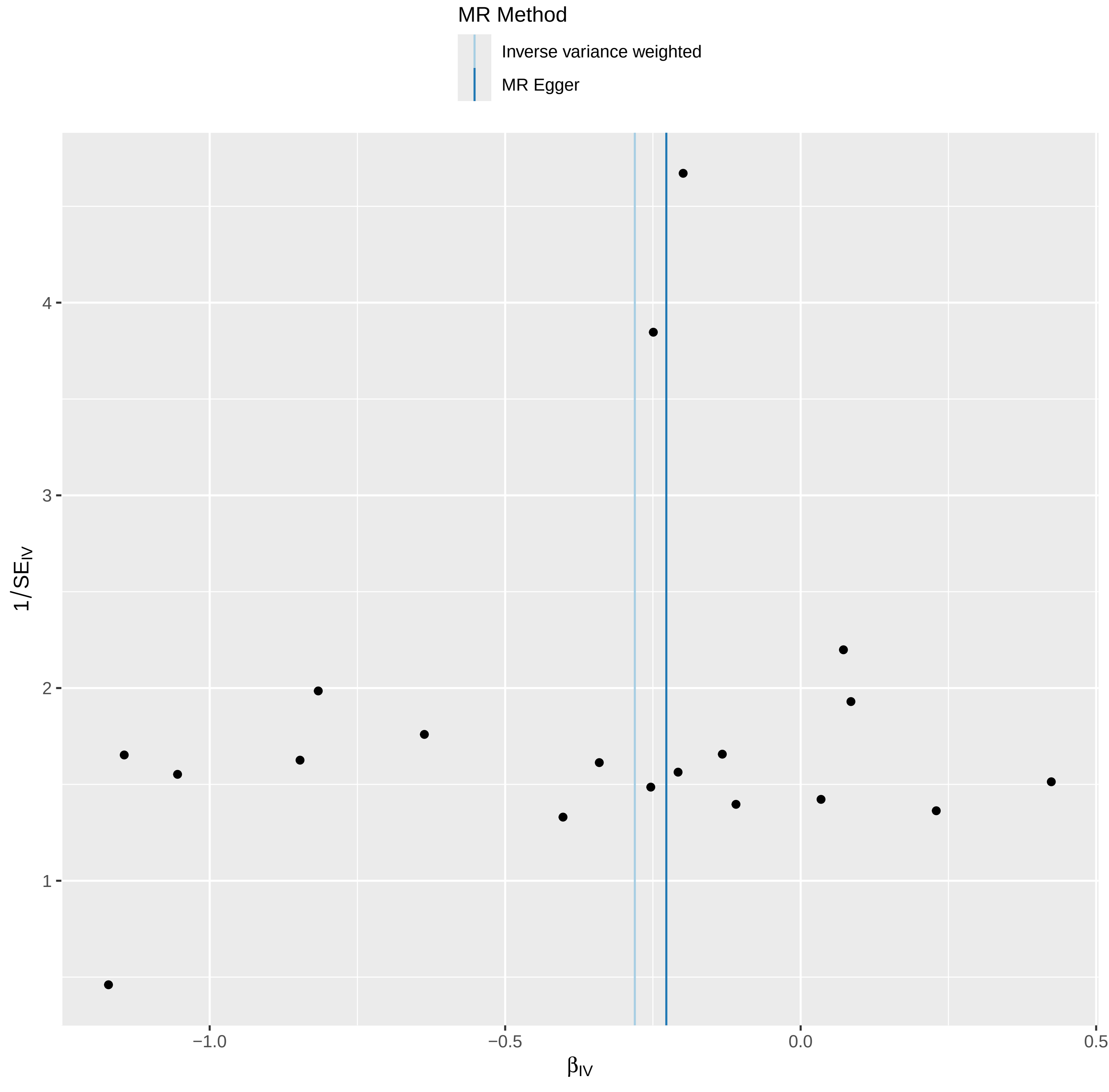

MR Method
Inverse variance weighted
MR Egger
4
3
2
1
1/SE_IV
−1.0
−0.5
0.0
0.5
β_IV

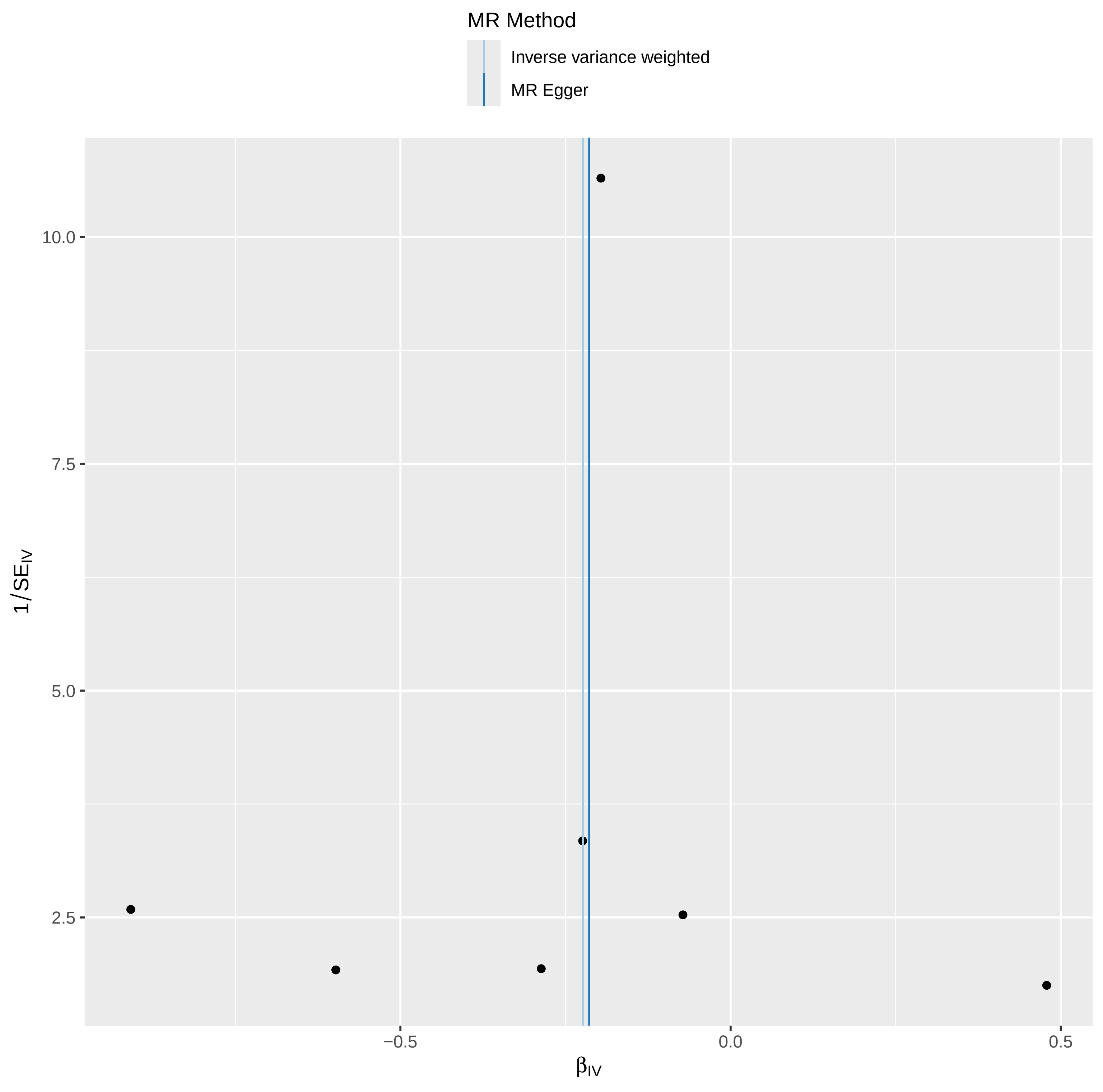
MR Method
Inverse variance weighted
MR Egger
10.0
7.5
5.0
2.5
1/SE$_{IV}$
−0.5
0.0
0.5
β$_{IV}$
