# Supplementary figures and images for: Circulating inflammatory cytokines and the risk of myasthenia gravis: a bidirectional Mendelian randomization study
Source: BMC Neurol. 2025 Jul 1;25:271. doi: 10.1186/s12883-025-04271-9 (PMC12211973; doi:10.1186/s12883-025-04271-9)

Sequence of pictures:ADA,CD40L,GDNF  
,IL-1 ,OPG,TNF-

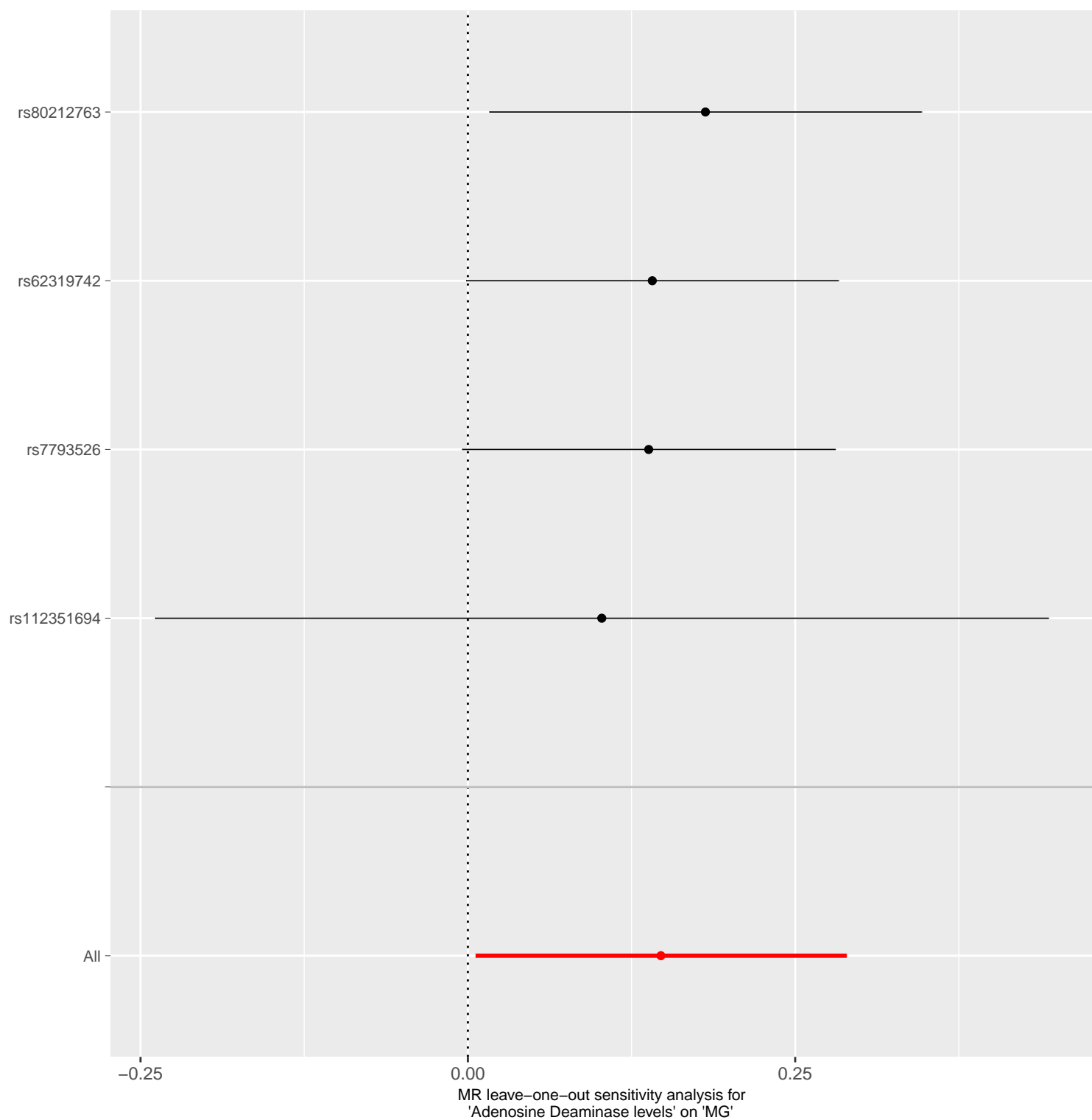

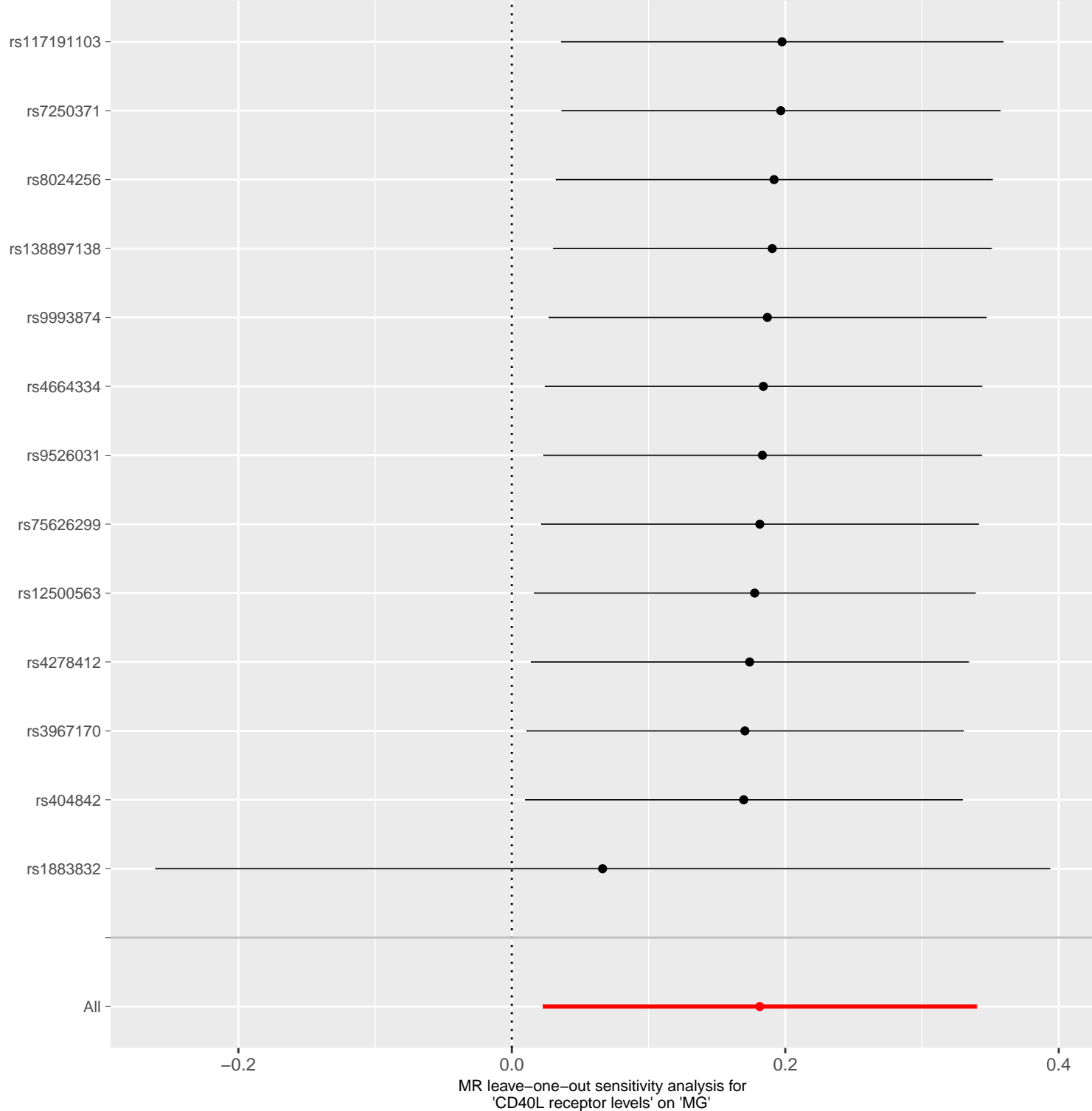

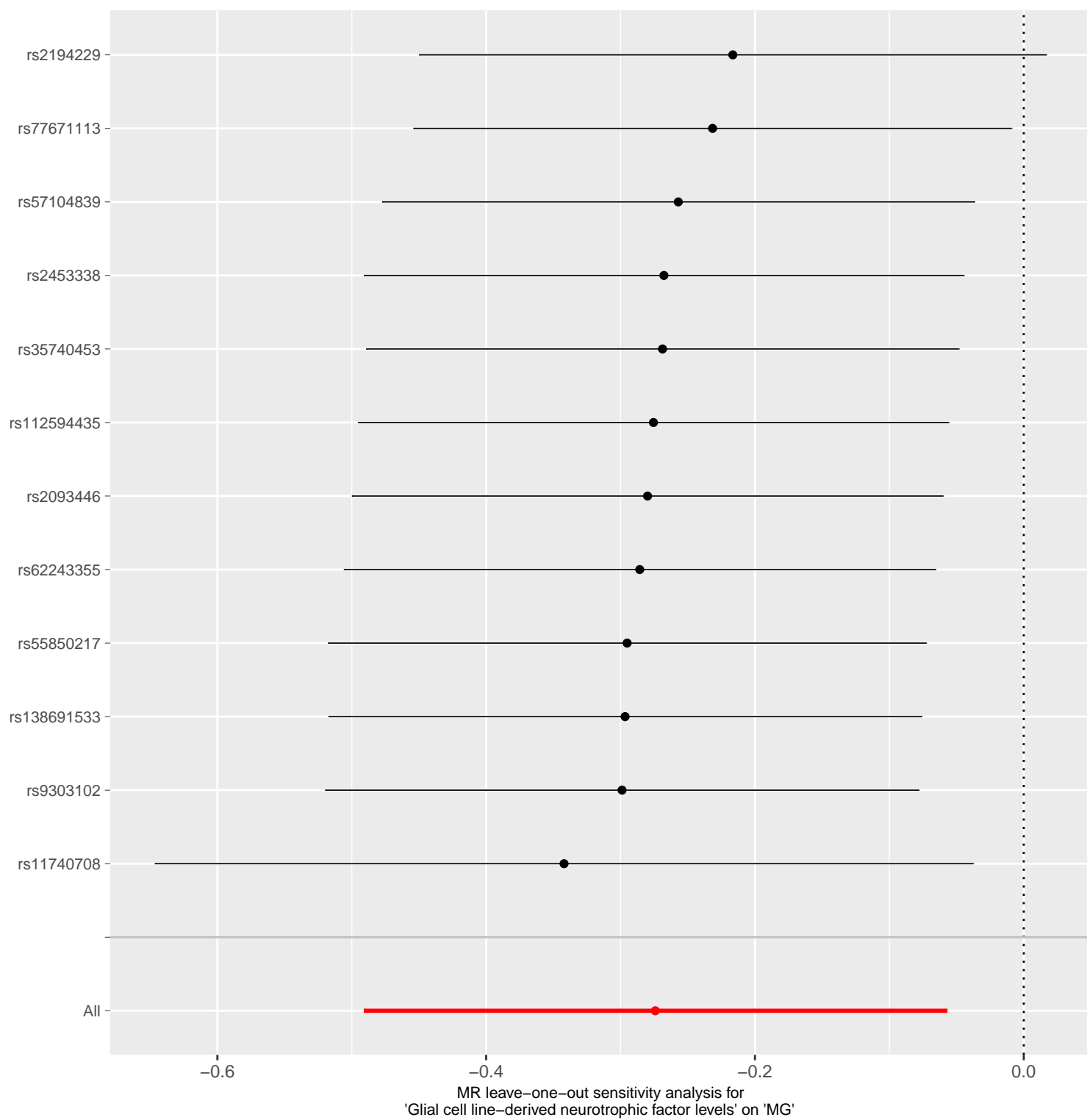

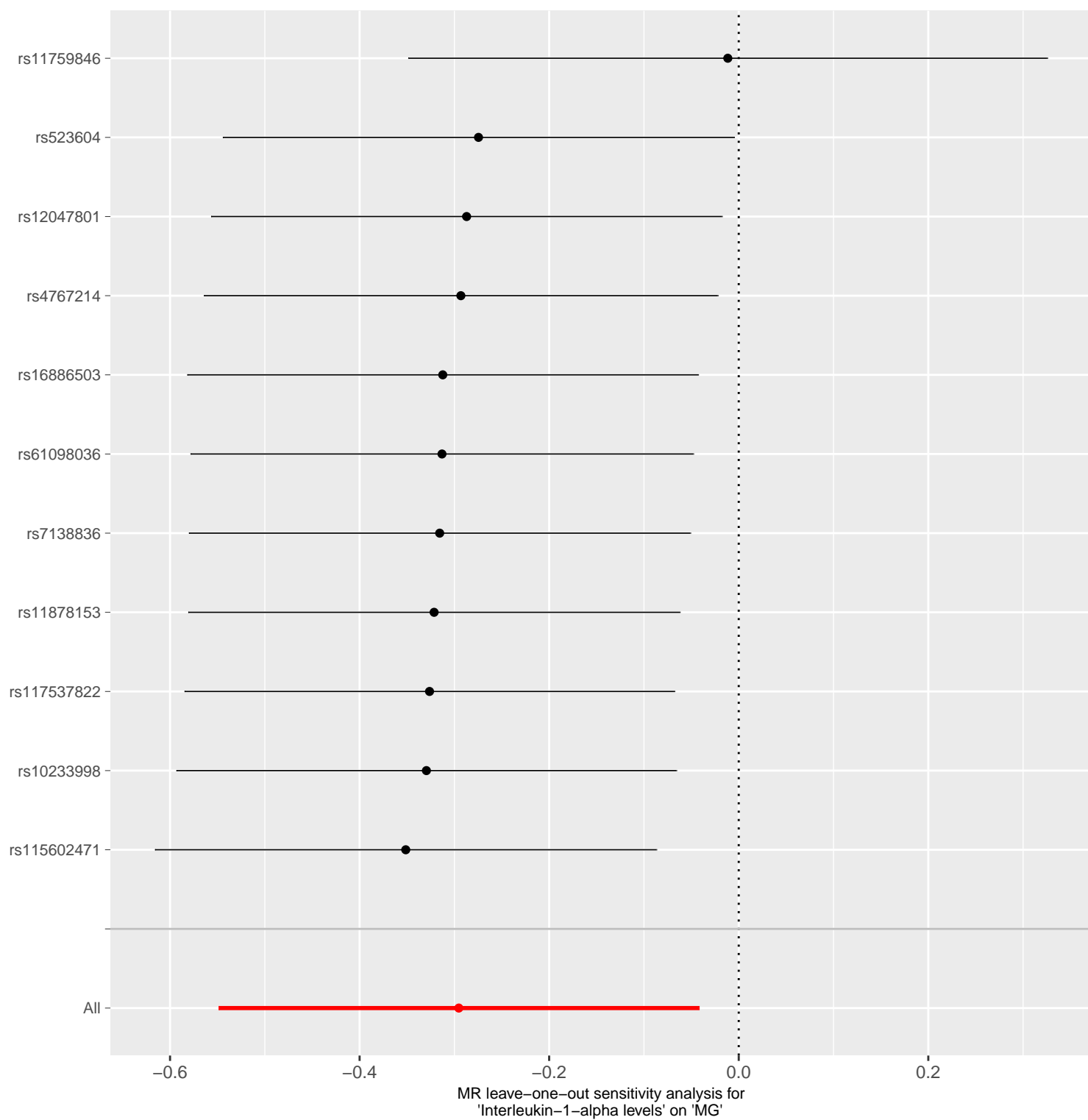

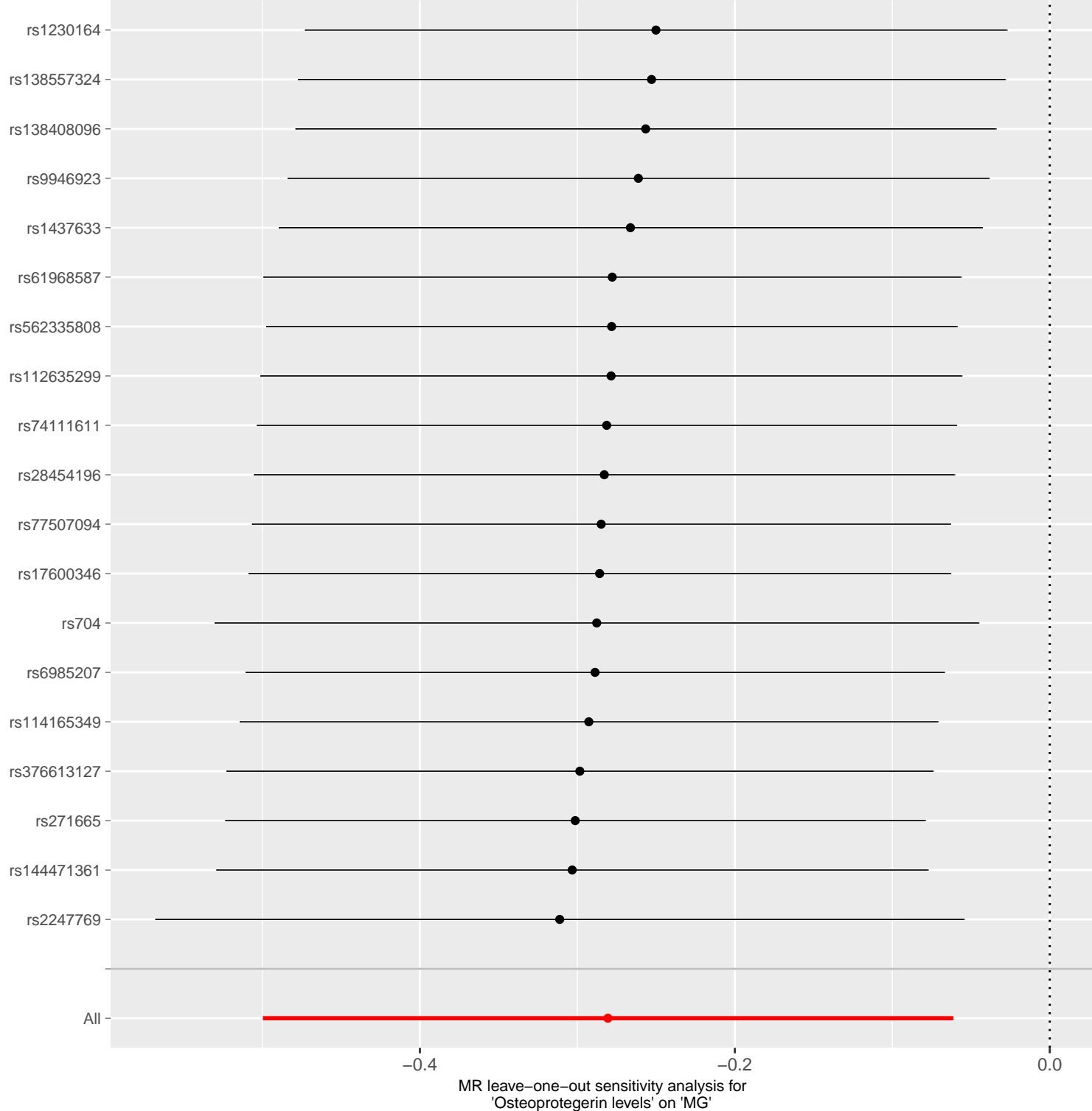

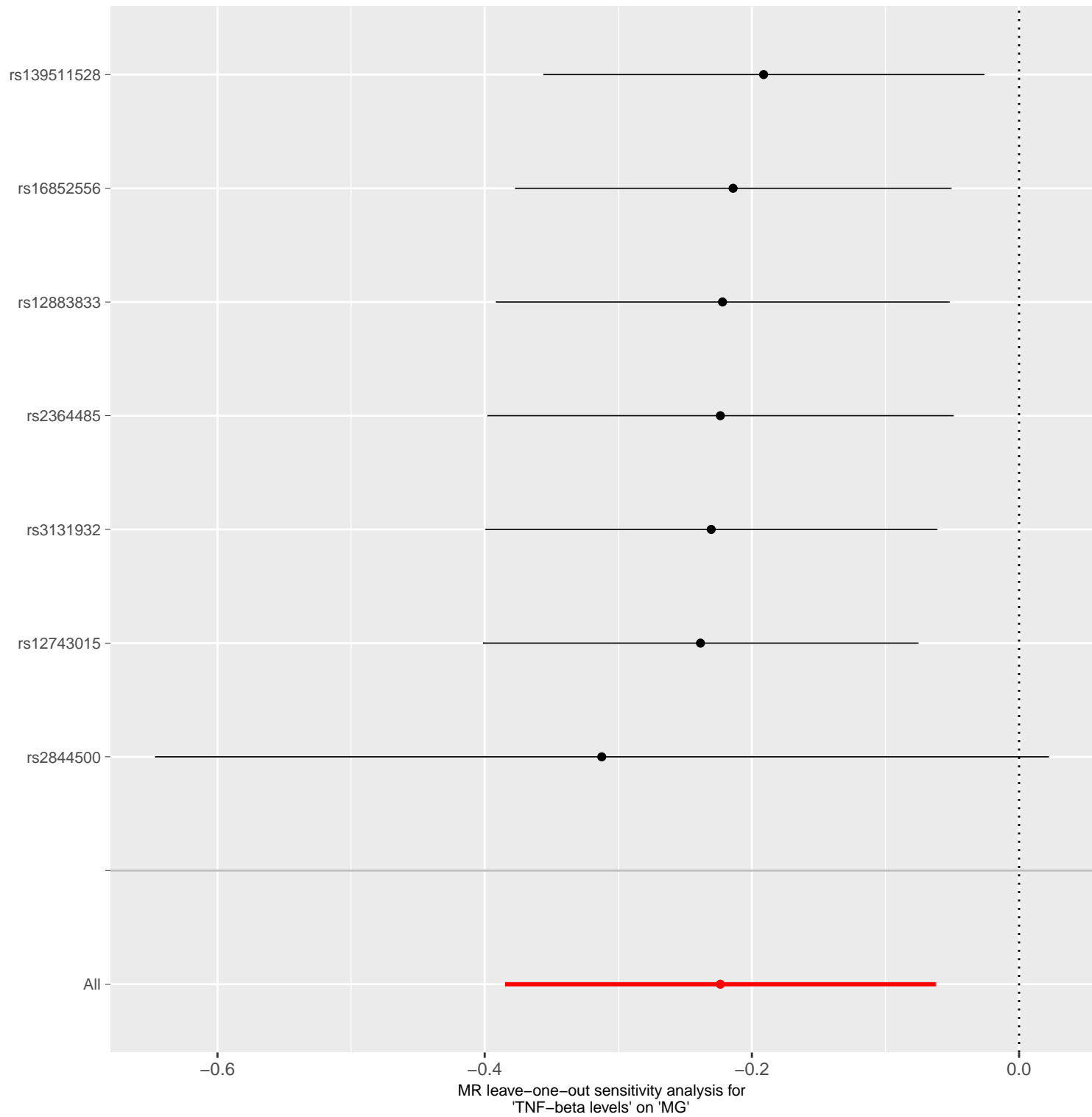

Supplement: Supplementary file 5 — Supplementary Material 5 [file 12883_2025_4271_MOESM5_ESM.pdf]

Sequence of pictures: CCL19、DNER、IL-12 、 IL-1  
、 MIP-1 、 TNF- and TRENCE

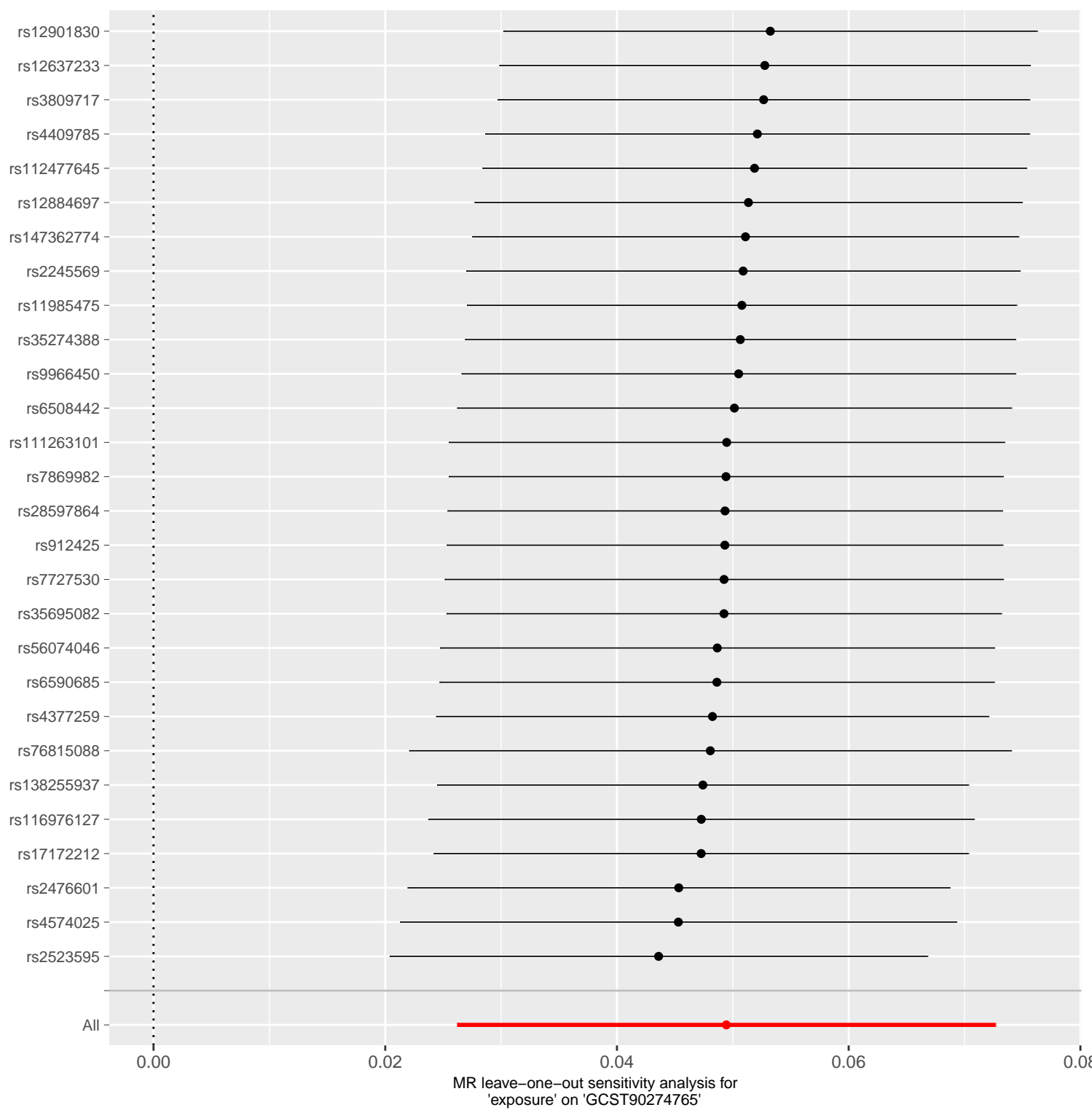

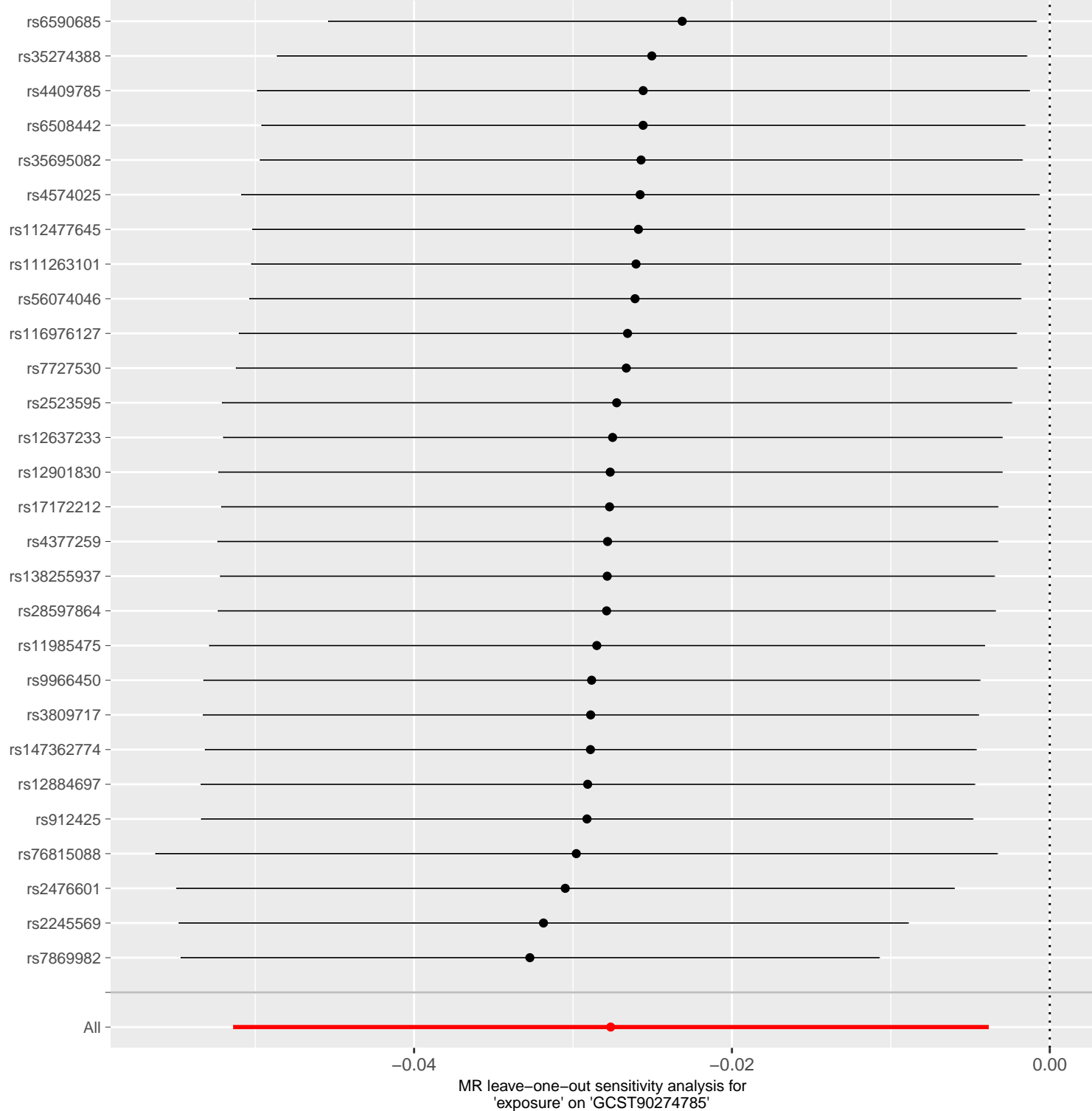

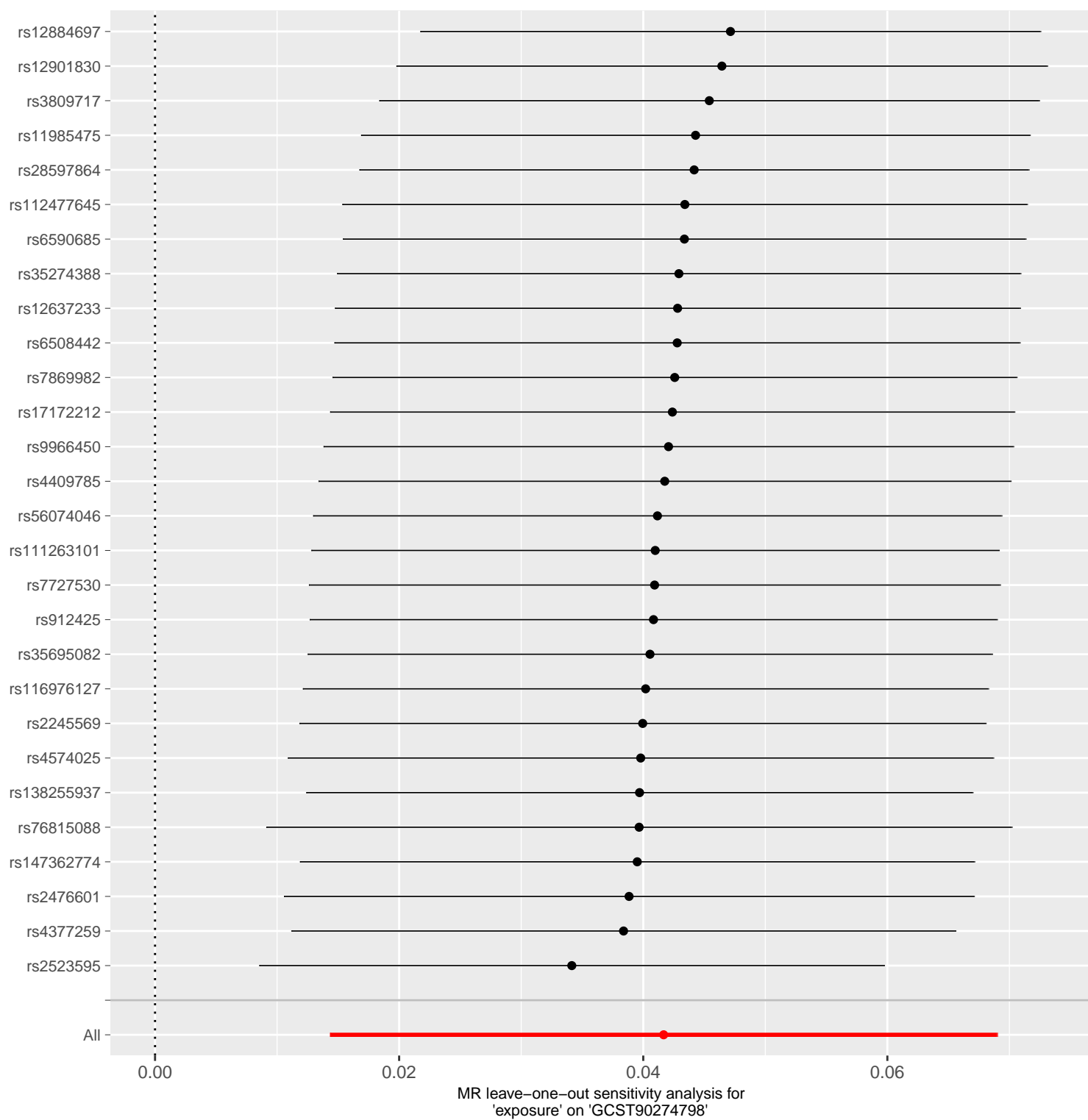

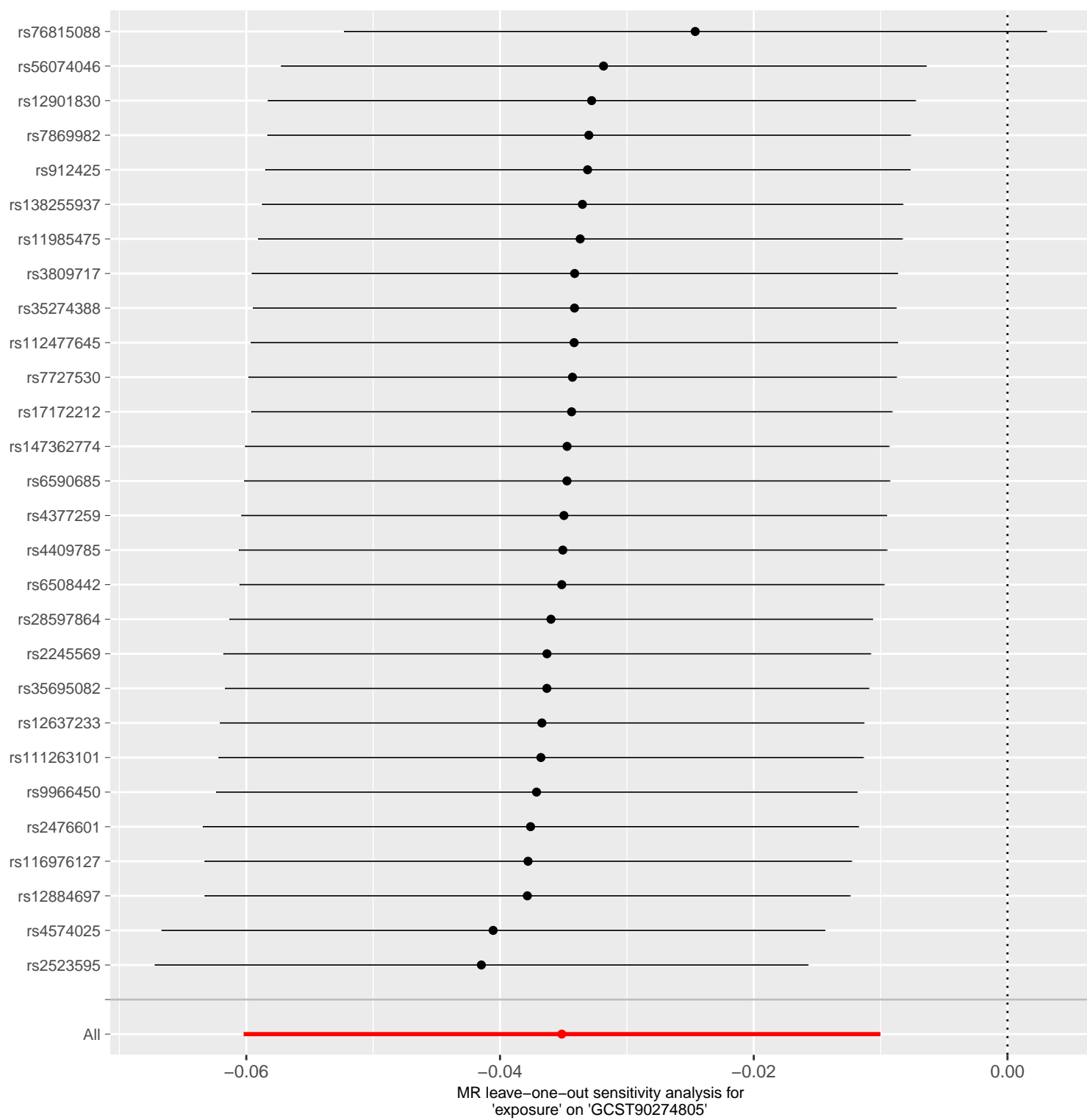

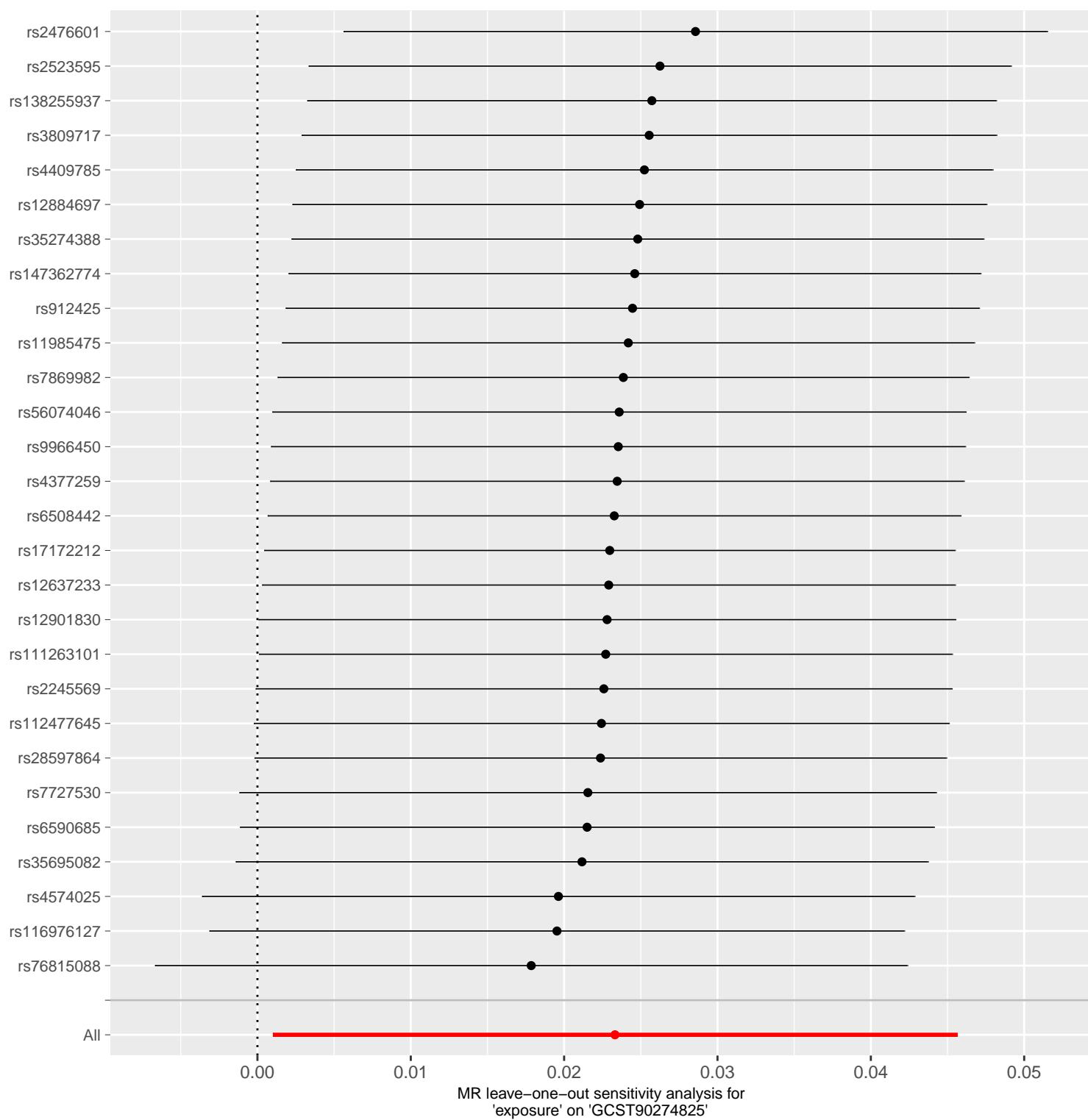

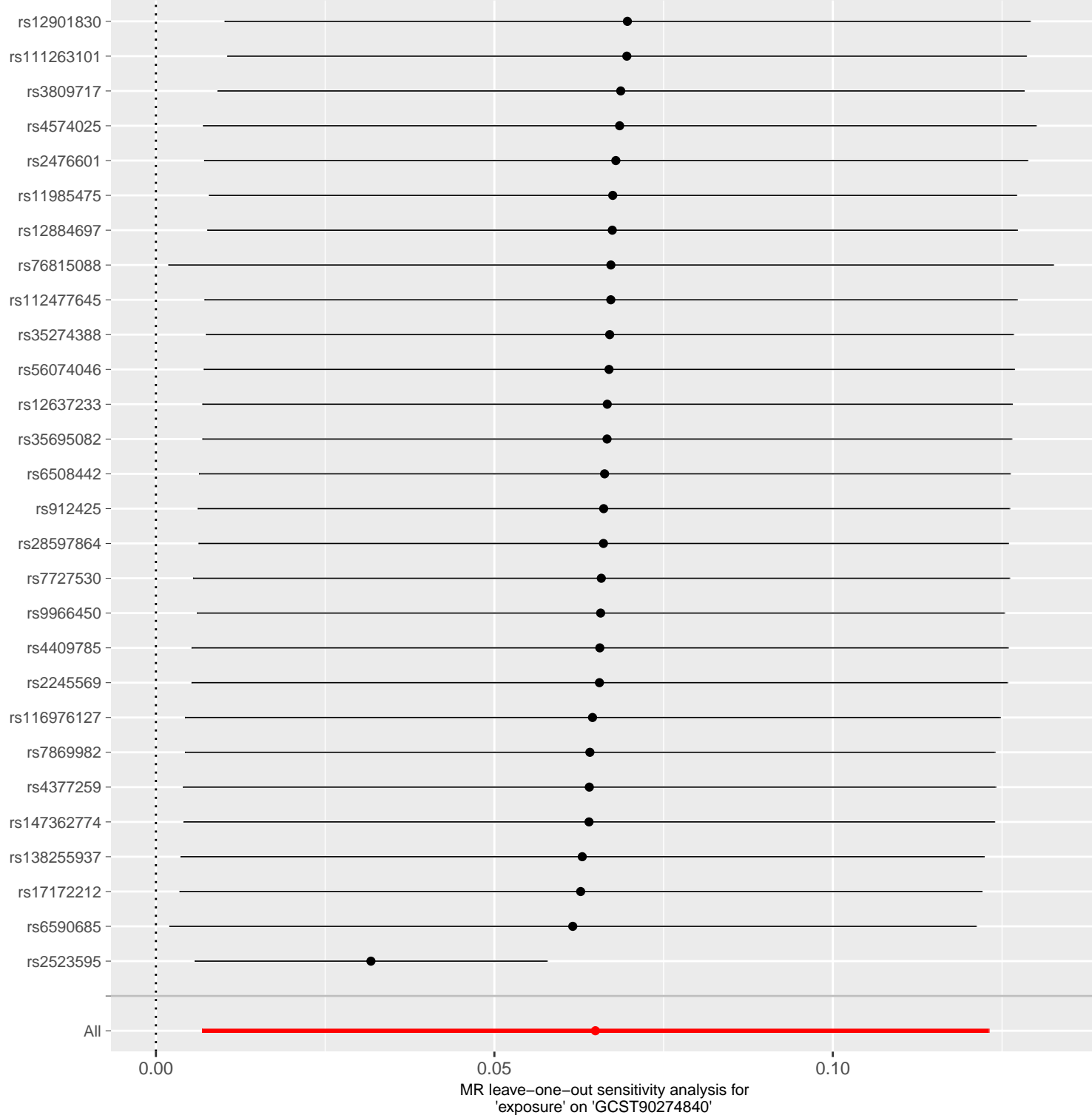

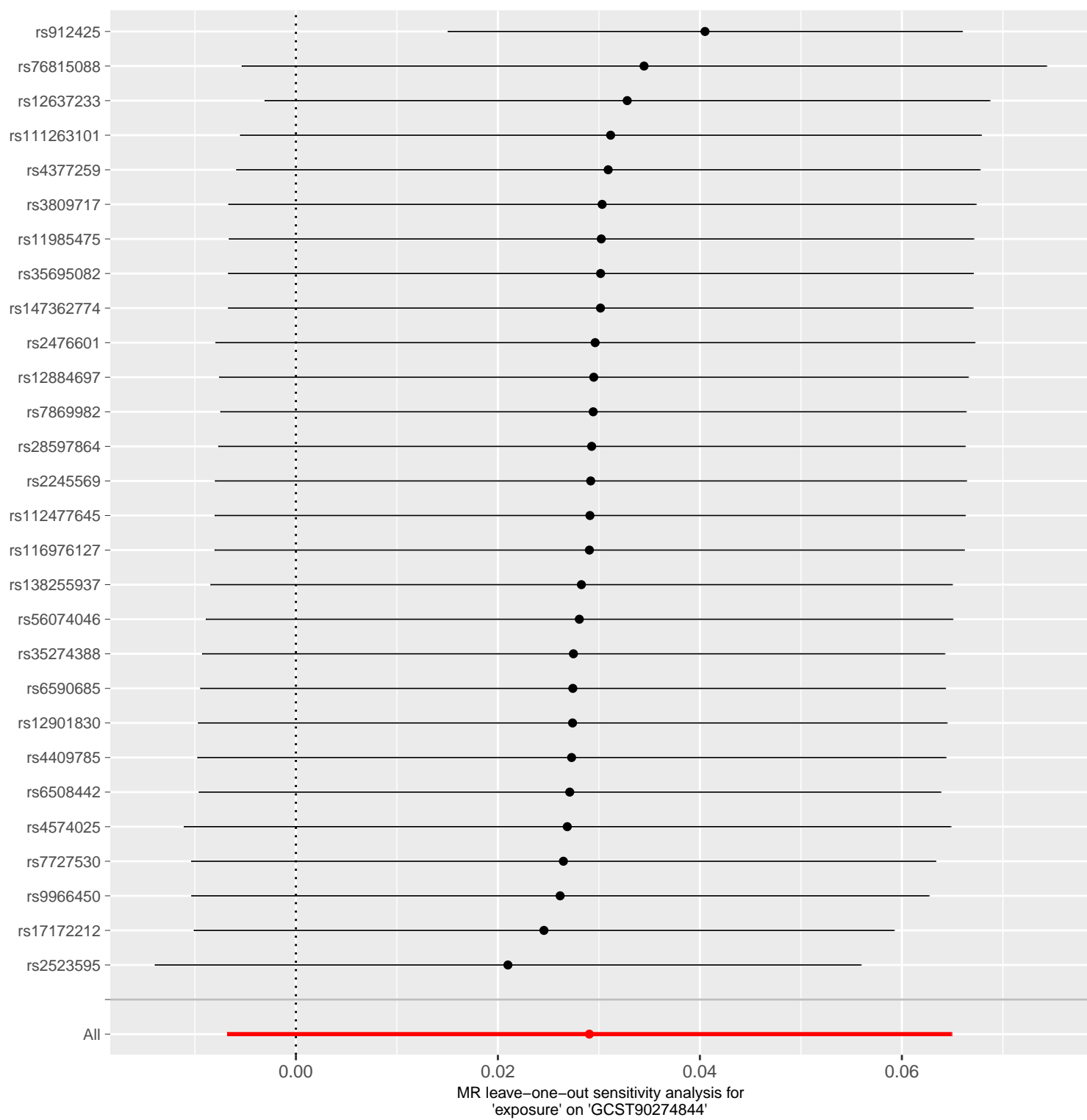

Supplement: Supplementary file 8 — Supplementary Material 8 [file 12883_2025_4271_MOESM8_ESM.pdf]
