# Supplementary material for: Circulating inflammatory cytokines and the risk of myasthenia gravis: a bidirectional Mendelian randomization study
Source: BMC Neurol. 2025 Jul 1;25:271. doi: 10.1186/s12883-025-04271-9 (PMC12211973; doi:10.1186/s12883-025-04271-9)
Supplement: Supplementary file 6 — Supplementary Material 6 [file 12883_2025_4271_MOESM6_ESM.pdf]

Sequence of pictures: CCL19、DNER、IL-12β、IL-1α、MIP-1α、TNF-β and TRENCE

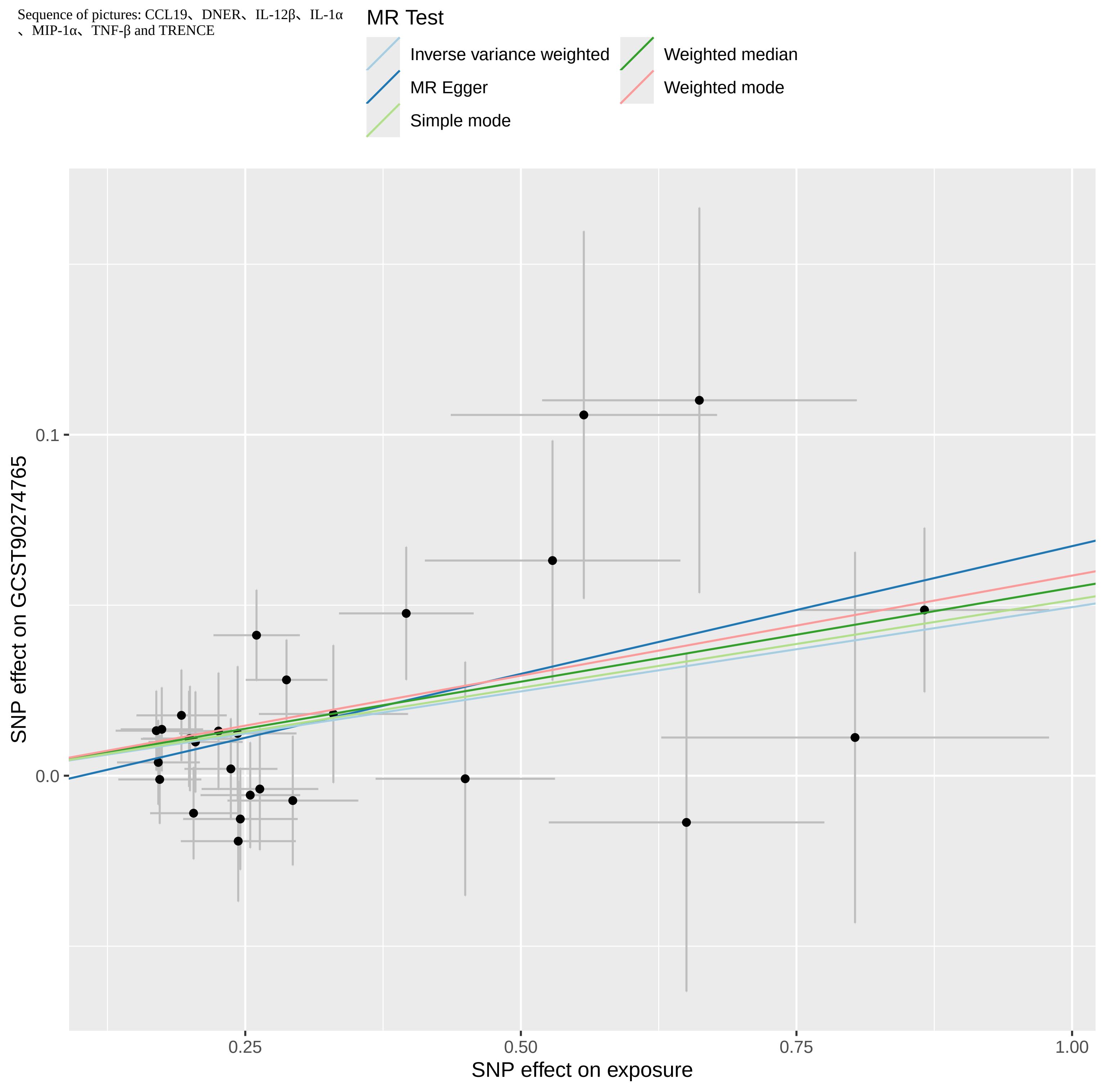

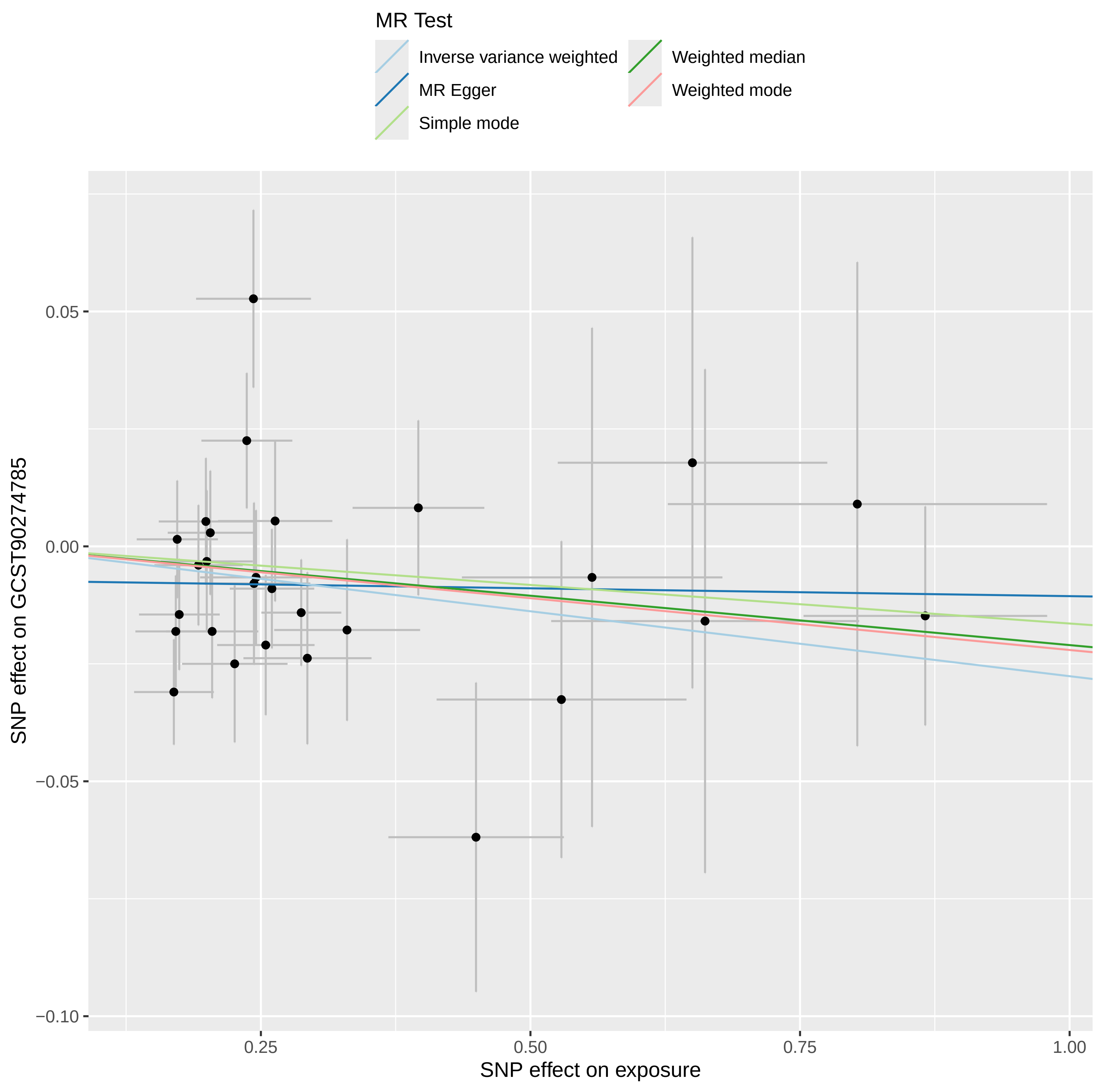

MR Test
Inverse variance weighted
MR Egger
Simple mode
Weighted median
Weighted mode
0.05
0.00
−0.05
−0.10
0.25
0.50
0.75
1.00
SNP effect on exposure
SNP effect on GCST90274785

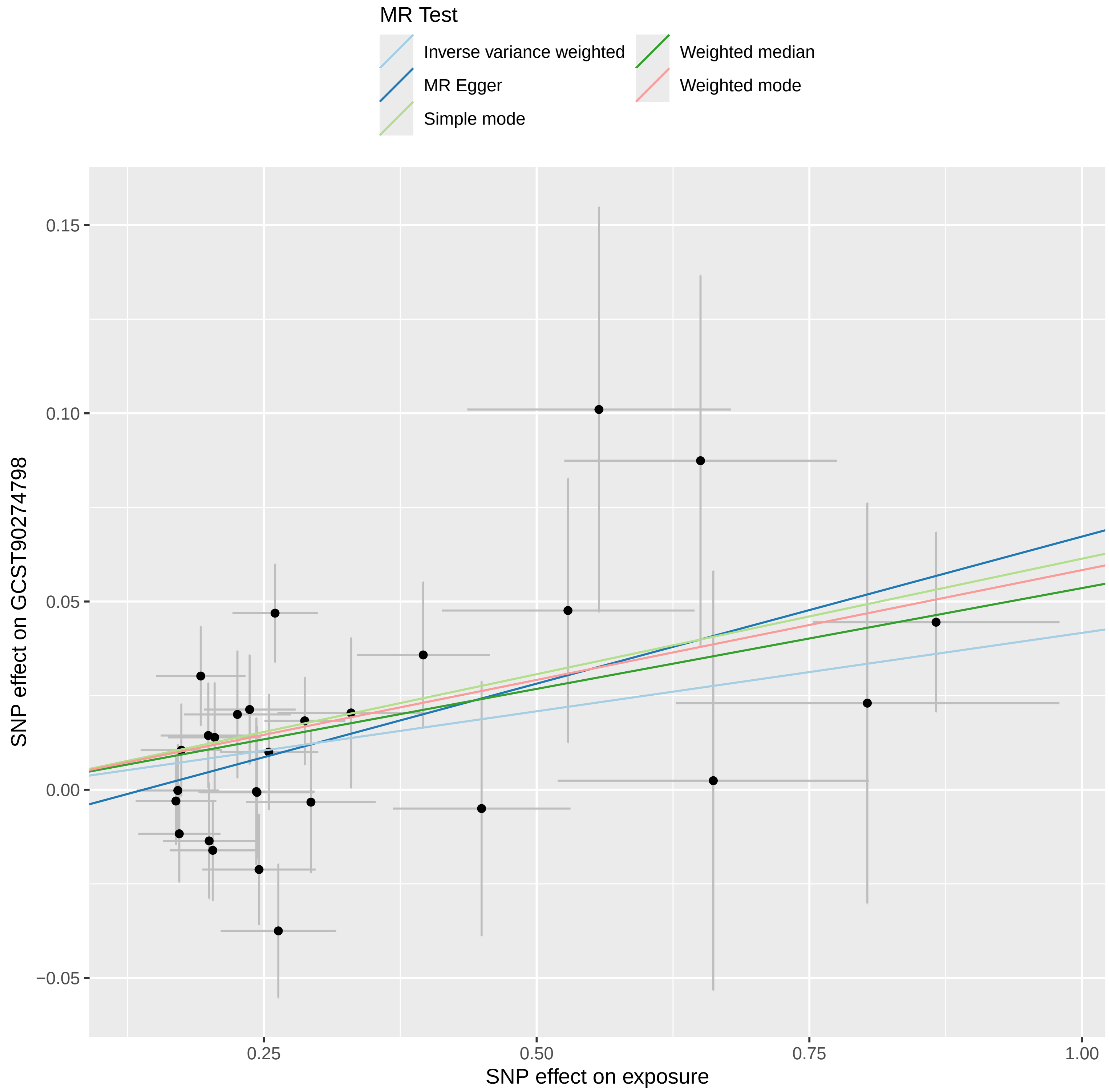
MR Test
Inverse variance weighted
MR Egger
Simple mode
Weighted median
Weighted mode
0.15
0.10
0.05
0.00
−0.05
SNP effect on GCST90274798
0.25
0.50
0.75
1.00
SNP effect on exposure

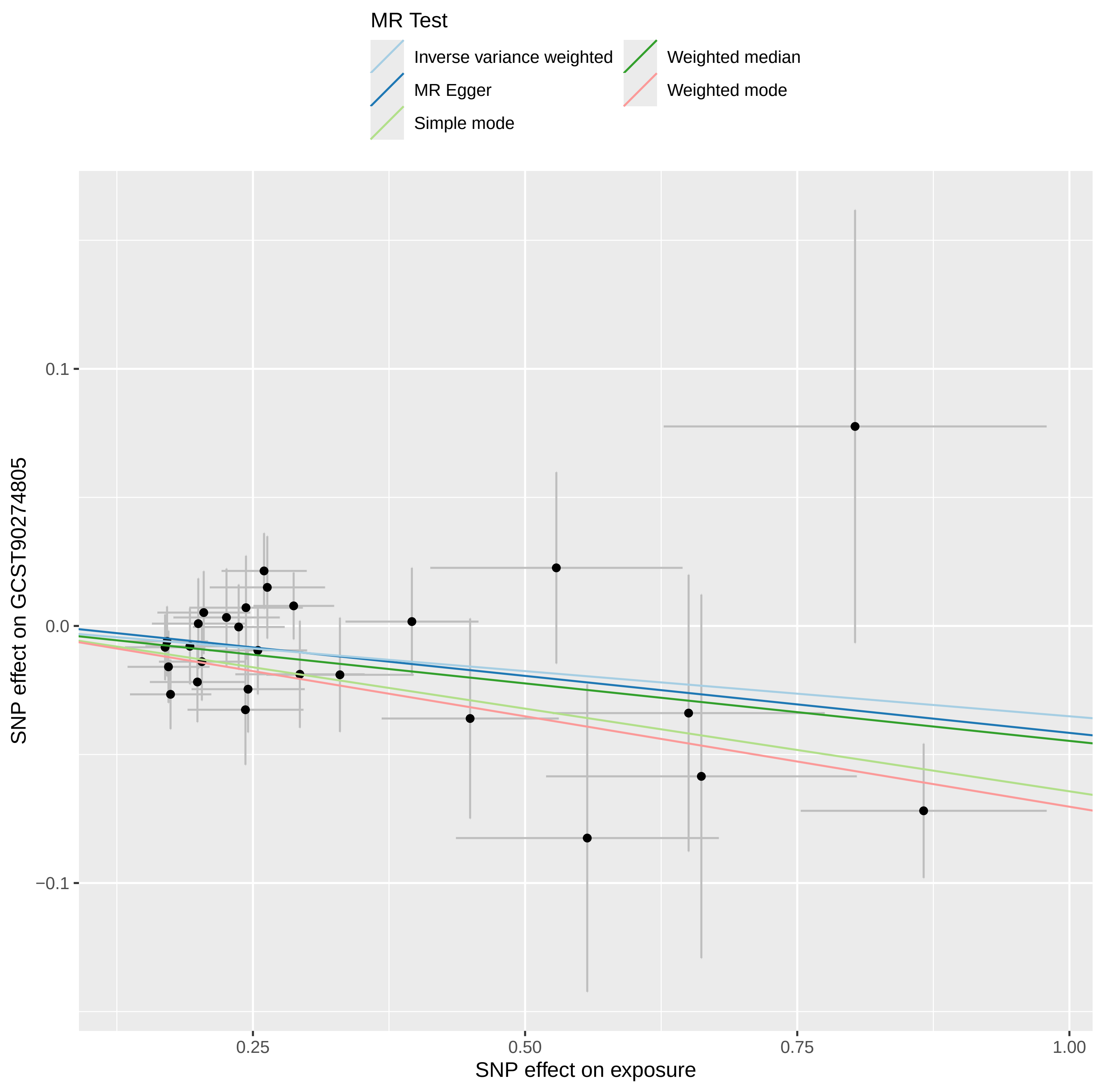
MR Test
Inverse variance weighted
MR Egger
Simple mode
Weighted median
Weighted mode
SNP effect on GCST90274805
0.1
0.0
−0.1
0.25
0.50
0.75
1.00
SNP effect on exposure

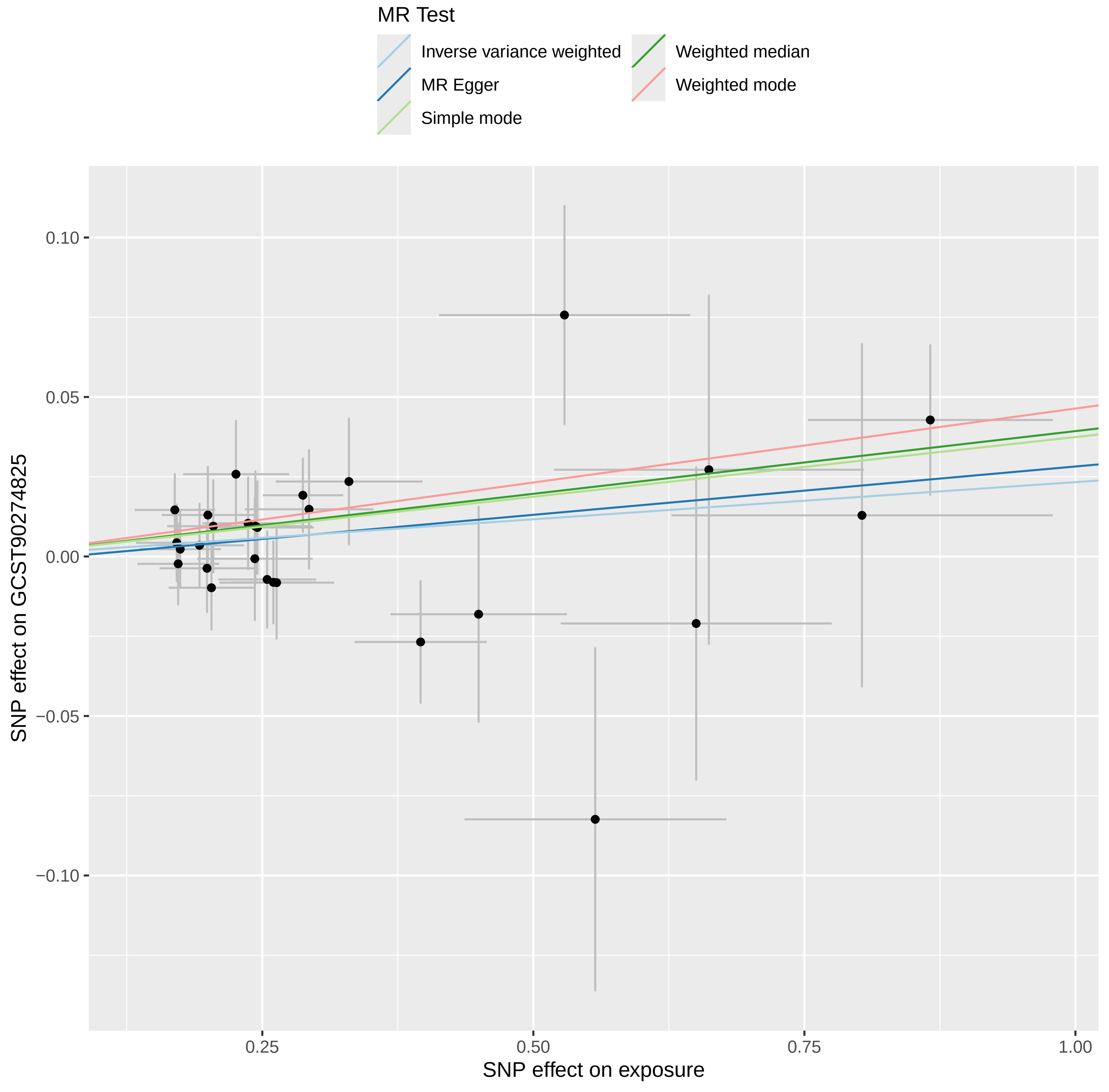
MR Test
Inverse variance weighted
MR Egger
Simple mode
Weighted median
Weighted mode
0.10
0.05
0.00
−0.05
−0.10
SNP effect on GCST90274825
0.25
0.50
0.75
1.00
SNP effect on exposure

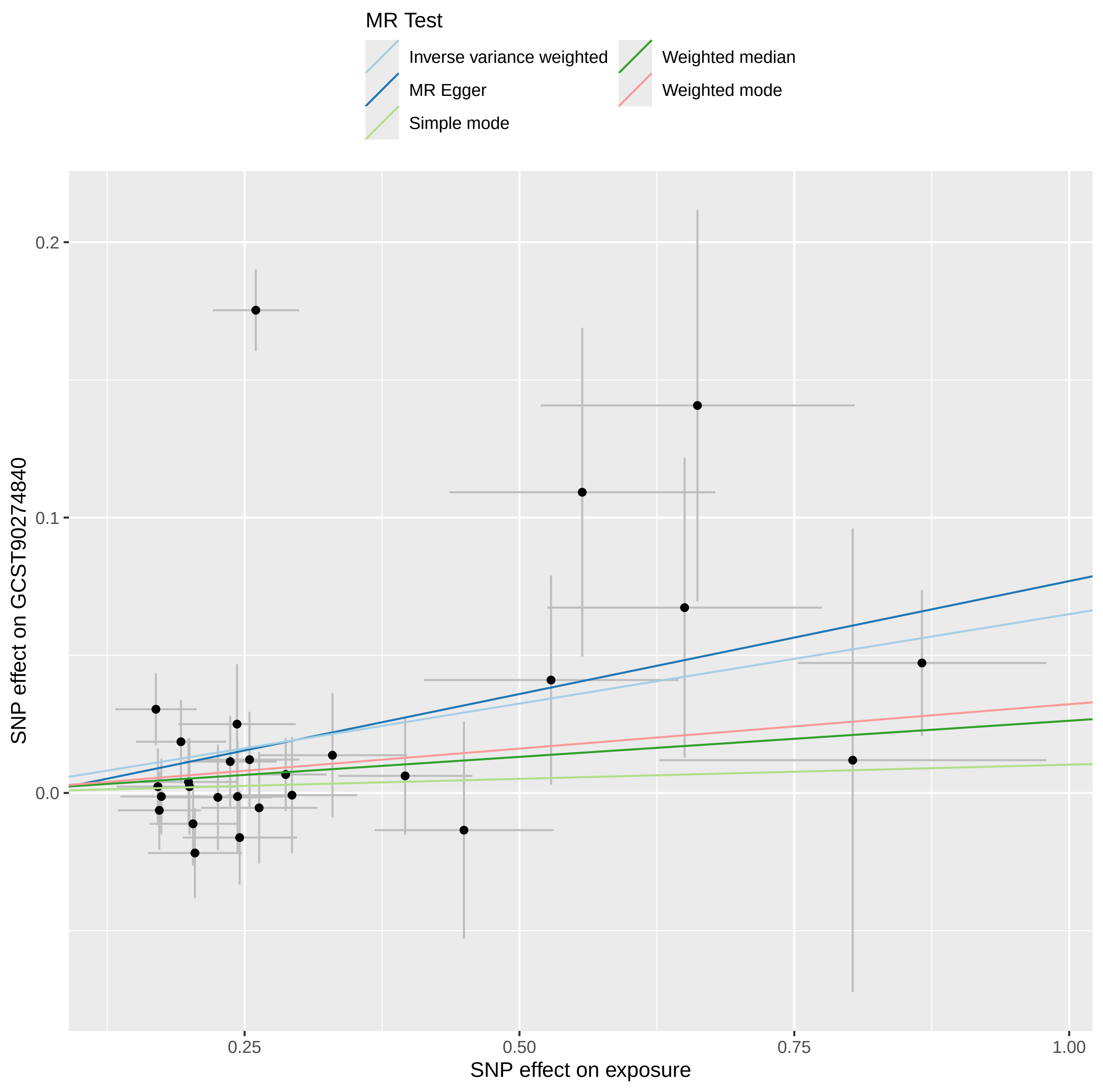
MR Test
Inverse variance weighted
MR Egger
Simple mode
Weighted median
Weighted mode
0.2
0.1
0.0
0.25
0.50
0.75
1.00
SNP effect on GCST90274840
SNP effect on exposure

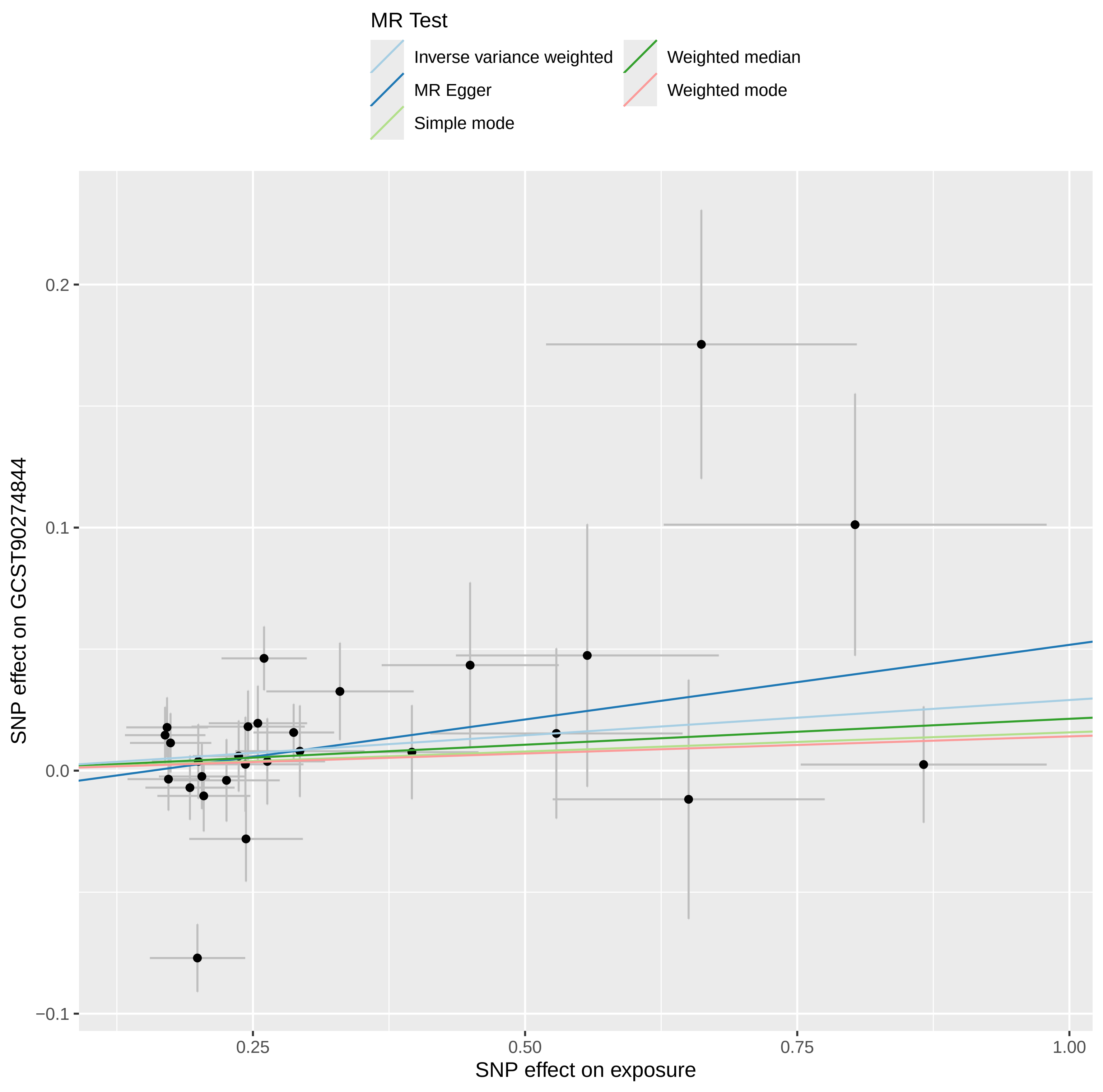
MR Test
Inverse variance weighted
MR Egger
Simple mode
Weighted median
Weighted mode
SNP effect on GCST90274844
0.2
0.1
0.0
−0.1
0.25
0.50
0.75
1.00
SNP effect on exposure
