# Supplementary material for: Circulating inflammatory cytokines and the risk of myasthenia gravis: a bidirectional Mendelian randomization study
Source: BMC Neurol. 2025 Jul 1;25:271. doi: 10.1186/s12883-025-04271-9 (PMC12211973; doi:10.1186/s12883-025-04271-9)
Supplement: Supplementary file 7 — Supplementary Material 7 [file 12883_2025_4271_MOESM7_ESM.pdf]

Sequence of pictures: CCL19、DNER、IL-12β、IL-1α
、MIP-1α、TNF-β and TRENCE

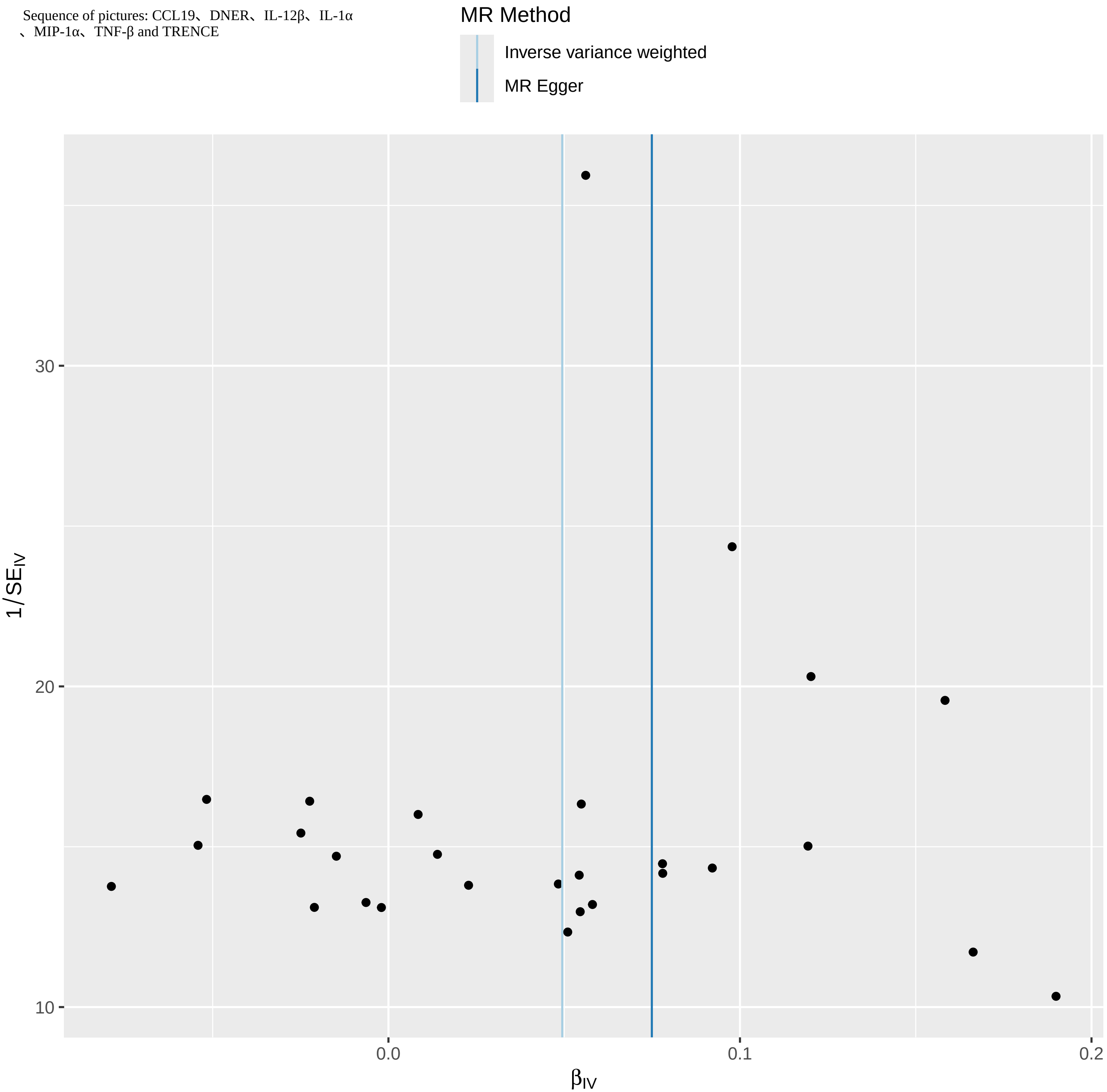

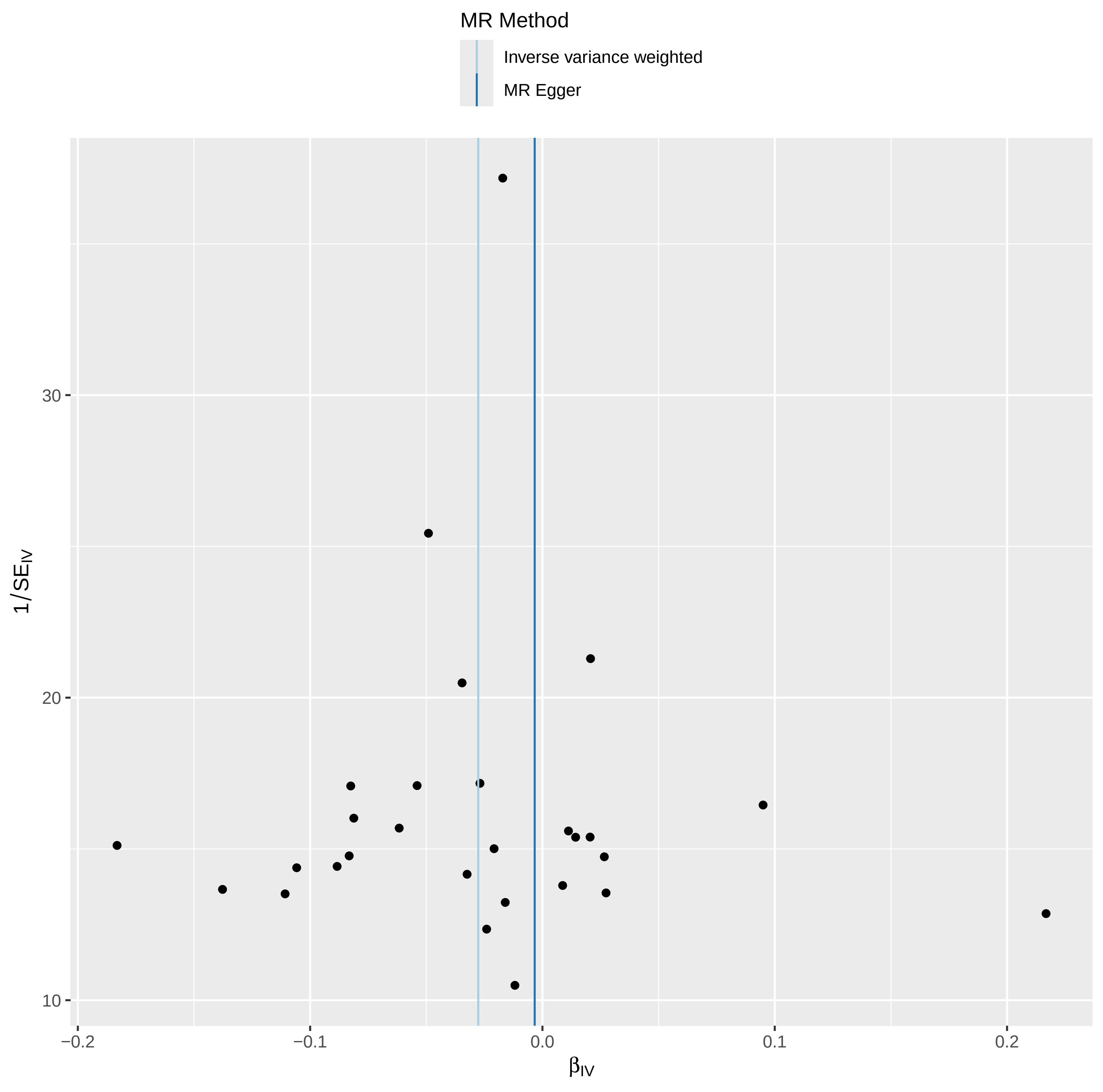
MR Method
Inverse variance weighted
MR Egger
1/SE_IV
β_IV
30
20
10
−0.2
−0.1
0.0
0.1
0.2

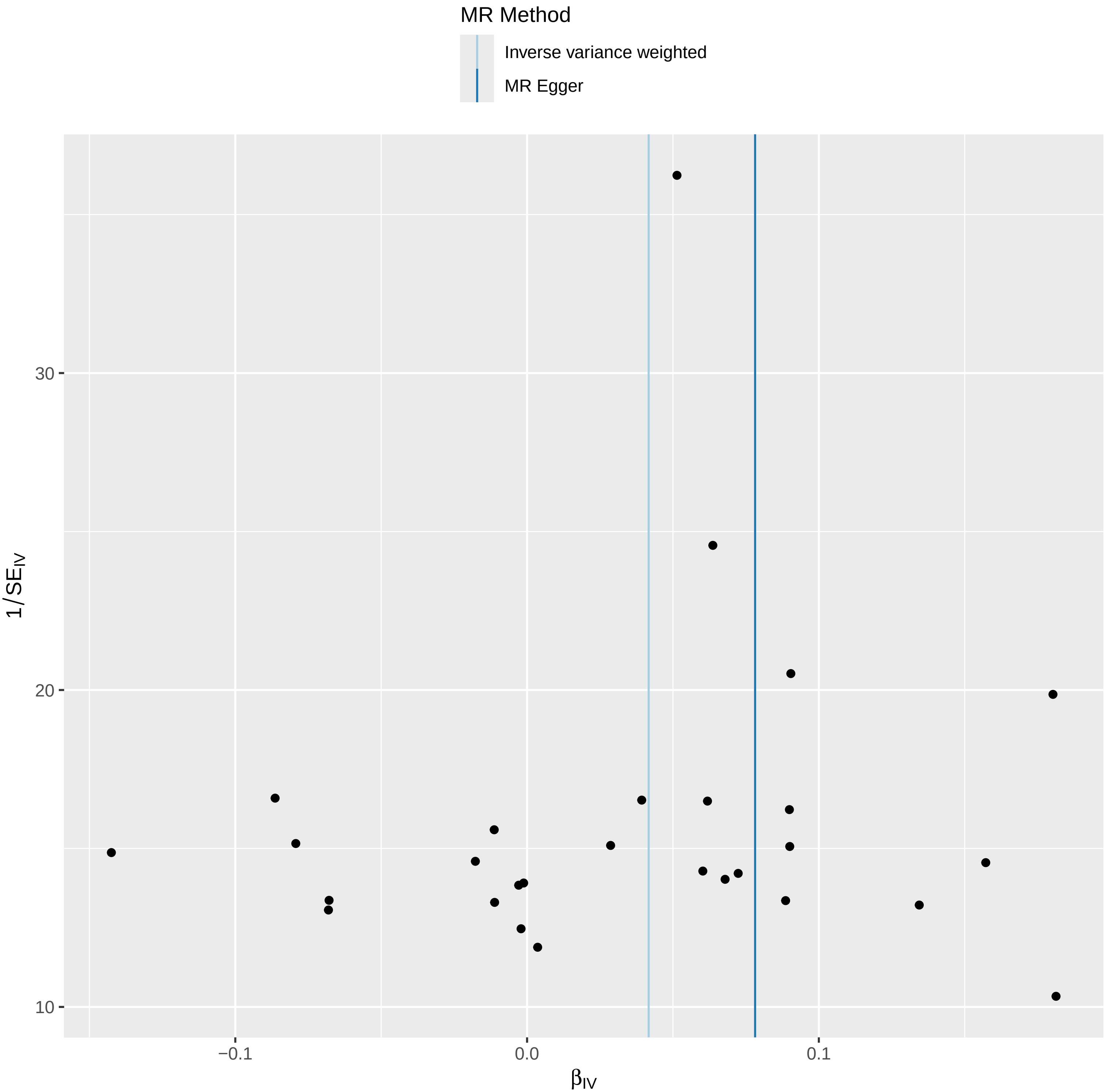
MR Method
Inverse variance weighted
MR Egger
30
20
10
1/SE_IV
−0.1
0.0
0.1
β_IV

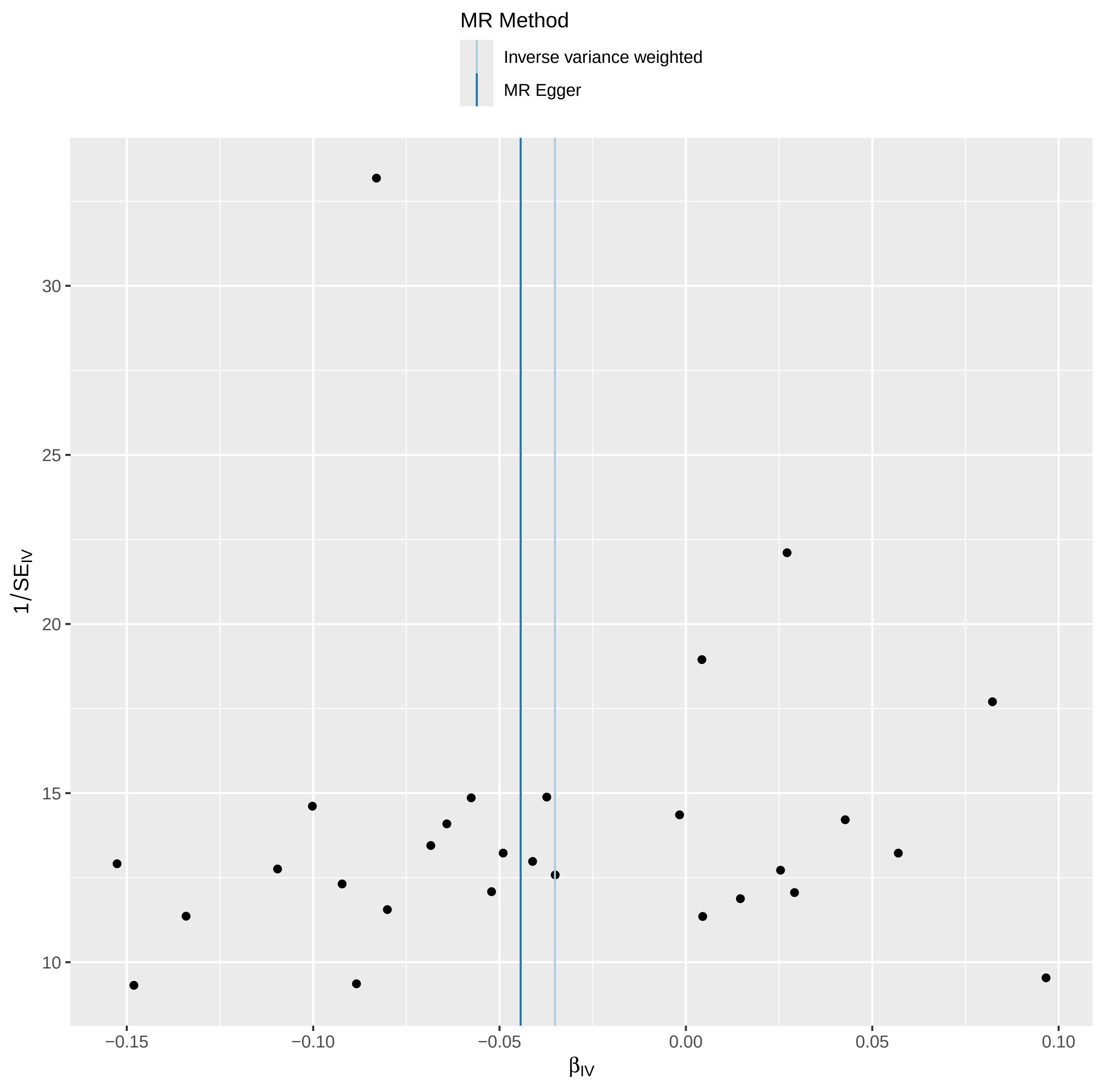
MR Method
Inverse variance weighted
MR Egger
1/SE_IV
β_IV
30
25
20
15
10
−0.15
−0.10
−0.05
0.00
0.05
0.10

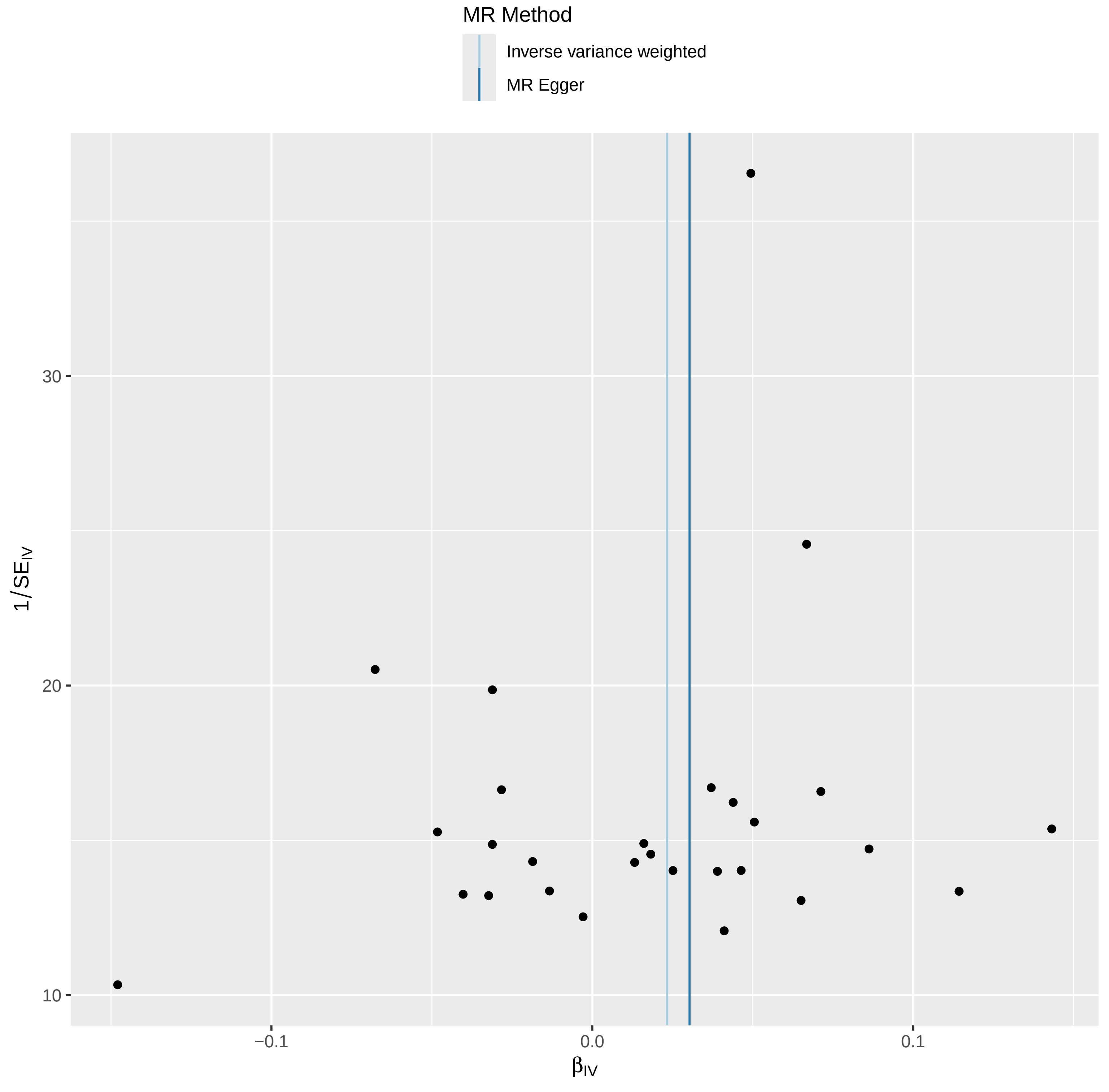
MR Method
Inverse variance weighted
MR Egger
1/SE_IV
30
20
10
−0.1
0.0
0.1
β_IV

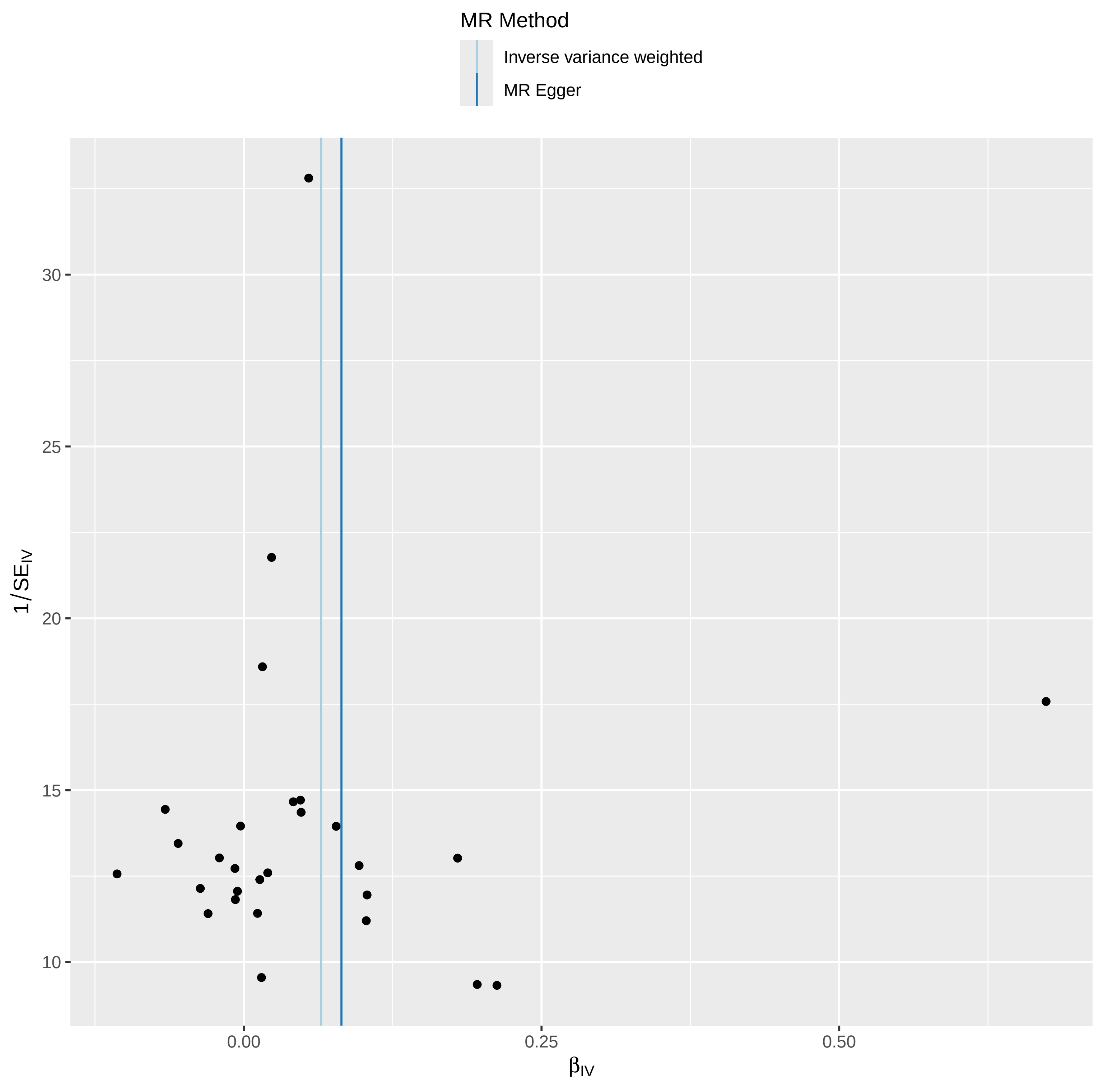
MR Method
Inverse variance weighted
MR Egger
30
25
20
15
10
1/SE_IV
0.00
0.25
0.50
β_IV

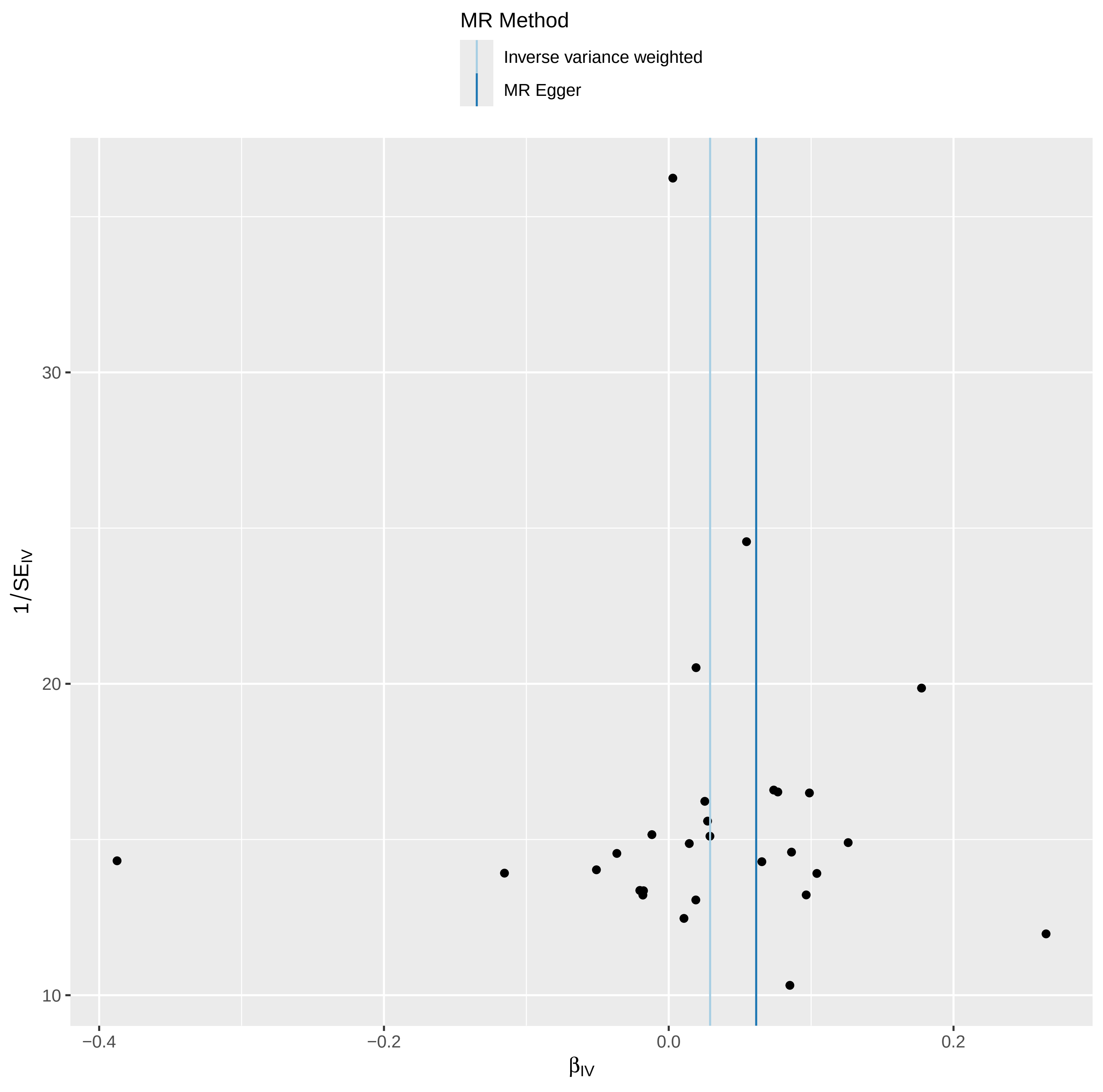

MR Method
Inverse variance weighted
MR Egger
30
20
10
1/SE$_{IV}$
−0.4
−0.2
0.0
0.2
β$_{IV}$
